# Rich dynamics and functional organization on topographically designed neuronal networks *in vitro*

**DOI:** 10.1016/j.isci.2022.105680

**Published:** 2022-11-26

**Authors:** Marc Montalà-Flaquer, Clara F. López-León, Daniel Tornero, Akke Mats Houben, Tanguy Fardet, Pascal Monceau, Samuel Bottani, Jordi Soriano

**Affiliations:** 1 Departament de Física de la Matèria Condensada, Universitat de Barcelona, E-08028 Barcelona, Spain; 2 Universitat de Barcelona Institute of Complex Systems (UBICS), E-08028 Barcelona, Spain; 3 Laboratory of Neural Stem Cells and Brain Damage, Institute of Neurosciences, University of Barcelona, E-08036 Barcelona, Spain; 4 Laboratoire Matière et Systèmes Complexes, Université de Paris, UMR 7057 CNRS, Paris, France; 5 University of Tübingen, Tübingen, Germany; 6 Max Planck Institute for Biological Cybernetics, Tübingen, Germany

**Keywords:** Neuroscience, Cell biology, Neural networks

## Abstract

Neuronal cultures are a prominent experimental tool to understand complex functional organization in neuronal assemblies. However, neurons grown on flat surfaces exhibit a strongly coherent bursting behavior with limited functionality. To approach the functional richness of naturally formed neuronal circuits, here we studied neuronal networks grown on polydimethylsiloxane (PDMS) topographical patterns shaped as either parallel tracks or square valleys. We followed the evolution of spontaneous activity in these cultures along 20 days *in vitro* using fluorescence calcium imaging. The networks were characterized by rich spatiotemporal activity patterns that comprised from small regions of the culture to its whole extent. Effective connectivity analysis revealed the emergence of spatially compact functional modules that were associated with both the underpinned topographical features and predominant spatiotemporal activity fronts. Our results show the capacity of spatial constraints to mold activity and functional organization, bringing new opportunities to comprehend the structure-function relationship in living neuronal circuits.

## Highlights

• Spatial anisotropies in cell cultures can be imprinted through topography
• Neurons grown on PDMS topographical substrates exhibit rich collective activity
• Neuronal network dynamics and activity propagation depend on topography details
• Network functional connectivity captures structural traits imprinted by topography

## Graphical abstract

**Figure:**
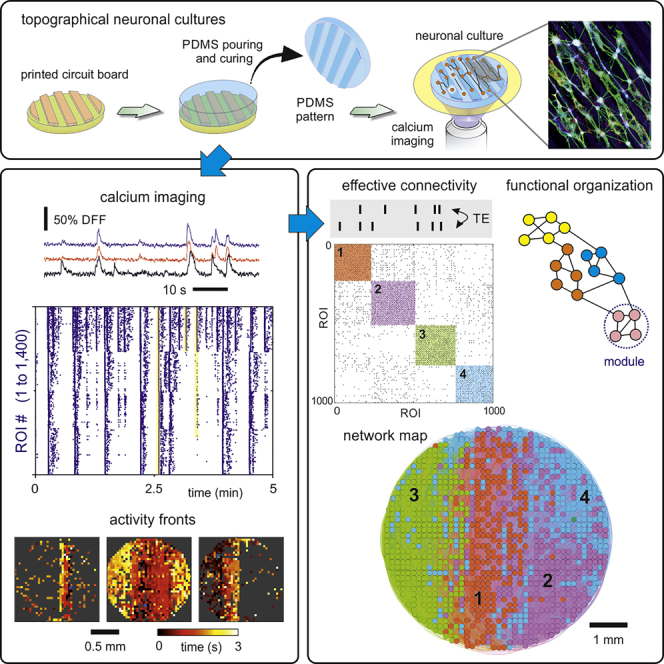

## Introduction

A fascinating yet intriguing property of living neuronal circuits is their capacity to exhibit a rich repertoire of activity patterns and functional states from a relatively hardwired structural architecture.^1^^,^^2^ This property is most prominent in the human brain, enabling the realization of precise and fast-changing tasks with precision, from motor action to memory and cognition,^3^^,^^4^ and that reveals the existence of intrinsic mechanisms and network traits for a swift dynamic reconfiguration of neural circuits. An established consensus is that modular and hierarchically modular network organization^5^ are fundamental hallmarks for the coexistence of diverse dynamic scenarios, allowing for both specialized computation at the scale of a module (functional segregation) and whole-network information exchange (functional integration)^6^^,^^7^ with balanced wiring efficiency cost.^8^

Modularity and integration-segregation balance are important actors in the functioning of neuronal circuits and play a key role in their robustness and flexibility.^4^ The sheer size of the brain and the intrinsic difficulty to monitor neuronal-level dynamics in detail, however, have fostered the development of *in vitro* preparations in which complex behavior at the mesoscale can be investigated.^9^^,^^10^^,^^11^ Culturing neurons in a controlled environment allows not only for an easy accessibility and manipulation of ∼100−1000 neurons but also for the design of true ‘structure-to-function’ laboratories to investigate the relation between physical wiring and emerging complex behavior.^12^^,^^13^^,^^14^ Two main techniques have excelled in the last decades to investigate emerging complex behavior in neuronal cultures,^15^ namely Calcium Imaging^16^ and multi-electrode arrays (MEAs).^15^^,^^17^ The former allows us to visualize changes in calcium concentration that occur upon neuronal activity with the aid of calcium fluorescence probes, whereas the latter directly measures the electrical signal of the neurons with superior temporal resolution. Modern technologies allow both techniques for non-invasive monitoring of relatively large networks (∼1000 neurons) for several days or weeks.

In the context of calcium imaging and MEAs, different experimental studies have pointed out the advantage of spatial constraints, connectivity guidance and modular designs^18^ to tune neuronal culture functionality^19^ and dynamics,^20^^,^^21^ or to facilitate the coexistence of segregated and integrated states.^22^ The advent of three-dimensional cultures have also paved the way toward elaborate ‘brain-on-chip’ devices that aim at reproducing and modeling *in vitro* the building blocks of complex brain behavior.^23^^,^^24^^,^^25^ Despite the importance of these neuroengineering efforts, it has been shown that an initially homogeneous distribution of neurons undergo significant reorganization that shape complex network features such as small-worldness and rich-club topology,^26^^,^^27^ whereas cultures exhibiting mild fluctuations in the spatial distribution of neurons in combination with activity-depend mechanisms evolve to exhibit modularity traits and balanced local-to-global connectivity.^28^^,^^29^

Although the above studies show that self-organization in neuronal circuits suffice to imprint rich functional traits, an aspect that remains unexplored is whether these traits can be accelerated or strengthened by incorporating coarse spatial constraints that break the isotropy of the substrate in which neurons grow. To advance in this quest, here we used mesoscopic neuronal cultures 6 mm in diameter grown on PDMS topographical substrates that contained elevations shaped as either parallel tracks or squares. Using effective connectivity and complex networks analyses we show that the underlying topography alters the way in which neurons develop and interconnect, shaping a rich repertoire of activity patterns and functional traits —most notably modularity— that contrast with the strongly coherent behavior and weak modularity of standard cultures grown on a flat surface. Our work provides compelling evidence that spatial constraints and structural features mold whole-network spontaneous activity and functional organization, opening new avenues for understanding the structure-function relationship in neuronal assemblies.

## Results

### PDMS topographical molds enrich spontaneous activity in primary neuronal cultures

**Figure 1:**
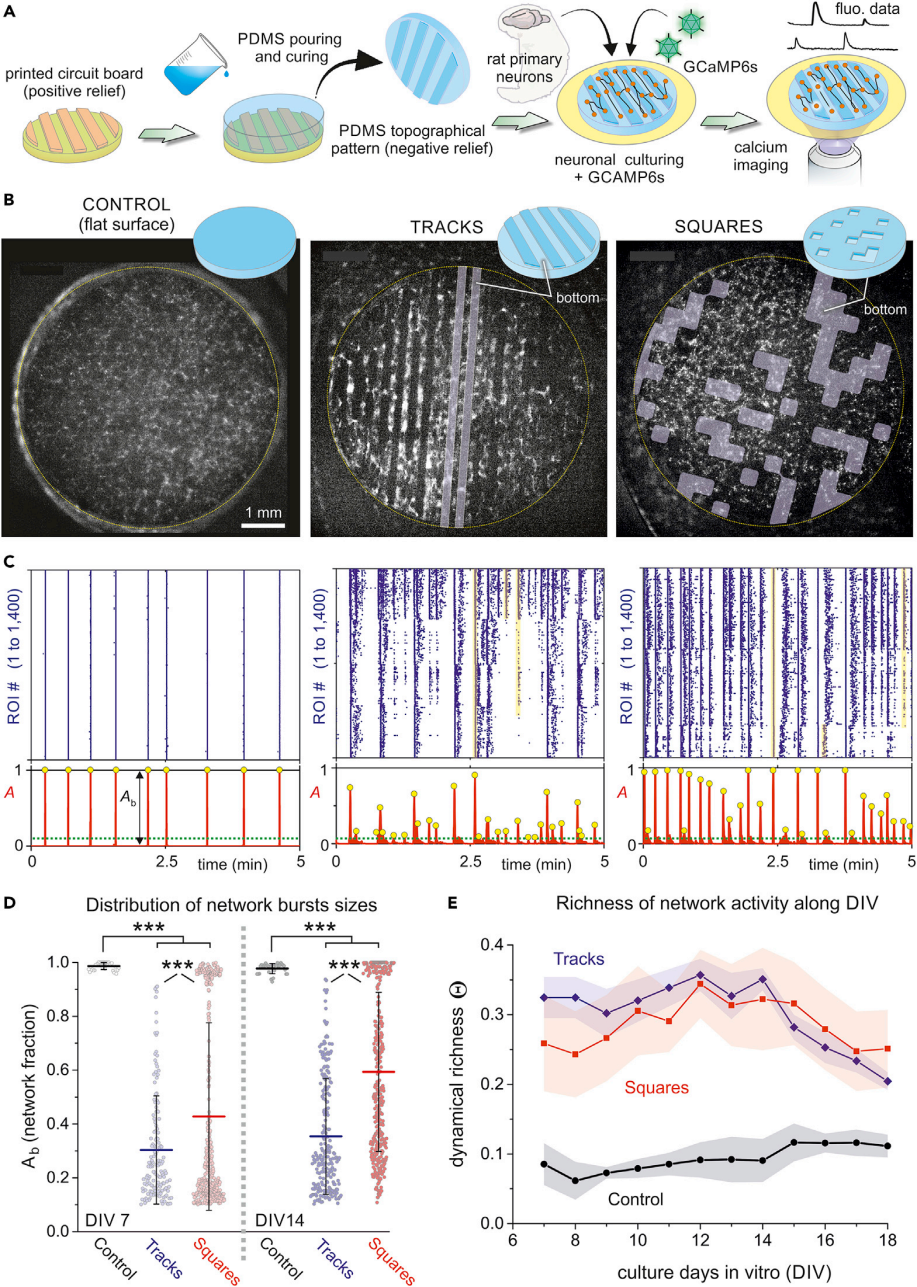
Spontaneous activity of neuronal cultures grown on PDMS topographical molds (A) Sketch of the experimental setup and procedure. A printed board circuit with a topographical relief 70 μm high (orange) was used as a master mold to pour and cure PDMS on it, leading to a design (blue) that is the negative relief of the original mold. Neurons were cultured on it in combination with GCaMP6s, delivered through adeno-associated viruses (AAVs). Spontaneous neuronal activity was then monitored through calcium fluorescence imaging. (B) Illustrative fluorescence images of the three studied topographical designs, namely a flat PDMS surface that serves as control (left), parallel tracks (center) and randomly positioned square valleys (right). All cultures were 6 mm in diameter and were recorded at DIV 14. Bright spots on the fluorescence images reveal active neurons. (C) Corresponding raster plots (top) and population activity *A* (bottom). Events encompassing more than 10% of the monitored Regions of Interest (ROIS) (green lines) were considered significant (yellow dots) and shaped ‘network bursts’ of size *A*_*b*_. Raster plots were ordered by ROIs similarity to highlight groups of coordinated activity. (D) Distribution of bursting sizes *A*_*b*_ for the three configurations and comparing young (day *in vitro*, DIV, 7) and mature (DIV 14) cultures. Data is plotted by pooling together all observations in n = 5 cultures for controls, 5 for tracks, and 6 for squares. ∗∗∗p < 0.001 (one-way ANOVA). (E) Dynamical richness Θ along development. Θ portrays the variability in the raster plots, which is much higher in topographical cultures, particularly in the range DIV 7–14. Data are represented as mean ± SD of the mean.

We used printed board technology to generate a master mold formed by copper motifs 70 μm high deposited on a fiberglass substrate (Figures 1A and S1). As described in Transparent Methods, the motifs were designed using computer-aided design software in combination with printed circuit board technology and included two main configurations, namely parallel copper tracks (termed *tracks*) and randomly positioned square copper blocks (*squares*). As sketched in Figure 1A, using copper tracks as example, the printed circuit board shaped a relief over which PDMS was poured and cured, giving rise to a topographical design that was the negative of the original mold. PDMS was then cut out as discs 6 mm in diameter that were firmly attached to a glass coverslip, and primary neuronal cultures from rat embryos were grown on the PDMS surface in a homogeneous manner. Cultures were later transduced with the genetically encoded calcium indicator GCaMP6s using adeno-associated viruses (AAVs) and spontaneous activity was monitored using calcium fluorescence imaging (Videos S1 and S2). Measurements on the same culture extended from day *in vitro* (DIV) 7, in which fluorescence signal was sufficiently strong for reliable analysis, to DIV 18, in which neurons started to degrade or detach from the PDMS surface.

Video S1. Spontaneous activity in a small region of the ‘tracks’ configuration at DIV 14, related to Figure 1 and 3 Spontaneous activity in a zoom-in region of the tracks configuration at DIV 14. Playback is 24x real time. At approximately recording time 54 s the processes (neurons and dendrites) in the culture can be appreciated. Several of these processes are aligned along the left track.

Video S2. Spontaneous activity in a small region of the ‘squares’ configuration at DIV 14, related to Figure 1 Spontaneous activity in a zoom-in region of the squares configuration at DIV 14. Playback is 6x real time.

Figure 1B provides illustrative fluorescence images of the prepared neuronal cultures (see also Videos S3 and S4). We included in our study control cultures plated on a flat PDMS, which provided a fundamental reference scenario to, first, investigate the capacity of PDMS patterning to suppress the persistent whole-network bursting of standard cultures, and second, to assess the impact of spatial anisotropies on network dynamics and functional organization. To emphasize the differences between controls and tracks/squares, in all analyses carried out here the behavior of control cultures is presented first.

**Figure 2:**
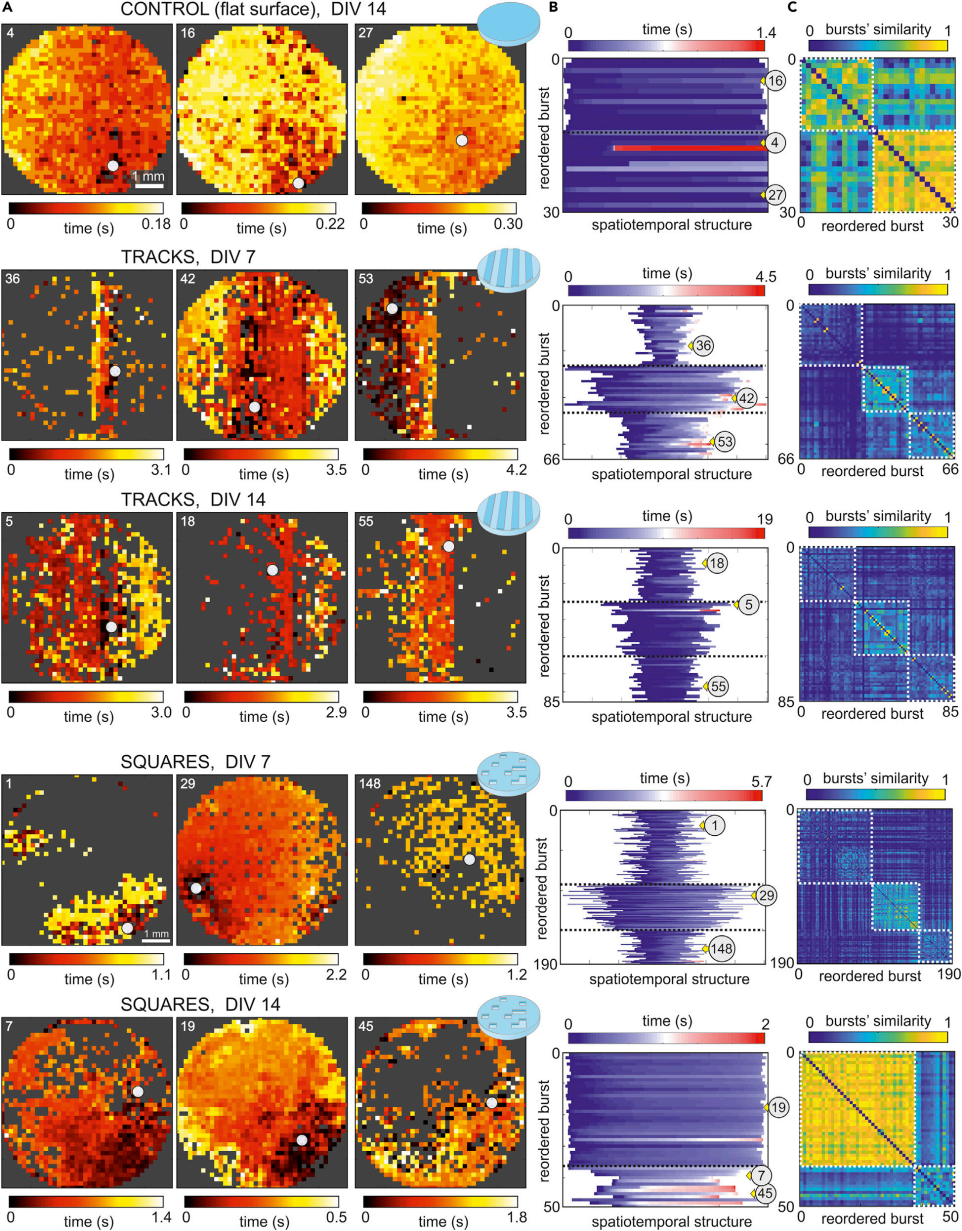
Repertoire of spatiotemporal patterns in control and topographical cultures (A) Representative examples of spatiotemporal activity fronts for controls, tracks and squares configurations. For the two latter, both young and mature developmental stages are shown for the same culture. Each colored dot in the image plots is an active ROI, with the color coded according to the time of activation (from black to yellow). Gray regions indicate absence of active ROIs. Activity in controls always comprises the entire network and propagates fast (Δt≃0.2−0.3 s). For lines and squares, activity switches between few sections of the culture or its whole extent, and much slowly (Δt≃1−3 s). The number on the top-left corner of each panel identifies the burst, whereas the big white dot signals the origin of activity. For the tracks configuration, the image plots are drawn for the topographical tracks to coincide with the vertical direction. For the squares configuration, the motifs are aligned with the image borders. (B) Classification of burst sizes and propagation. Each band corresponds to a burst which are ordered according to their similarity. The width of a band indicates the number of ROIs involved in the burst, whereas its color indicates the propagation time. Bands with similar color scheme portray bursts with akin spatiotemporal structure. The numbers within a gray circle indicate the position of the bursts represented in (A). The doted black lines separate the different groups of bursts. (C) Detailed classification in the form of similarity matrix and following the same organization as in (B). The brighter the color, the higher the similarity among bursts. White dashed boxes identify the groups of similar bursts.

Video S3. Spontaneous activity in a full 6 mm diameter cultures with ‘tracks’ topography at DIV 14, related to Figures 1, 2 and 4 Spontaneous activity for a ‘tracks’ culture at DIV 14 in which its entire extent (6 mm diameter PDMS disc) visualized. The video plays at approximately 5x speed and covers 2 min of actual recording. Different structures of spatiotemporal fronts can be observed, e.g., extending half culture or two tracks. Contrasting velocities of activity propagation along or across tracks can be also appreciated.

Video S4. Spontaneous activity in a full 6 mm diameter culture with ‘squares’ topography at DIV 7, related to Figures 1 and 2 Spontaneous activity for a ‘squares’ culture at DIV 7 in which in which its entire extent (6 mm diameter PDMS disc) visualized. The video plays at approximately 5x speed (covering 2 min of actual recording) for a better appreciation of the activity fronts and their propagation. The video is related to Figs. 1 and 2.

To quantify the collective behavior of the prepared cultures, we recorded spontaneous activity in each configuration for 30 min, to next extract the fluorescence traces in small Regions of Interest (ROIs) that contained 5–10 neurons each and that covered uniformly the area of the culture (Figure S2), giving rise to about 1,400 ROIs.

The recorded fluorescence data was analyzed to extract the activation time of each ROI, and data represented in the form of raster plots. Figure 1C shows 5 min of representative data for the cultures depicted in Figure 1B. Activity in control cultures was characterized by episodes of highly coherent behavior in which all ROIs activated together in a short time window of ∼200 ms (*network bursts*) or remained practically silent. *Tracks* and *squares* configurations, by contrast, showed a much richer dynamic repertoire, in which network bursts of different sizes coexisted (yellow bands in Figure 1C). Network bursts extended longer periods of time (on the order of seconds) for these topographical configurations, and sporadic activity outside bursts was also more abundant.

The rich variety of network burst sizes was reflected in the population activity A, which counts the fraction of ROIs that coactivate together (Figure 1C, bottom panels). Networks bursts whose size were above background activity (typically 10% of A) were considered significant and denoted $A_{b}$. Although all events exhibited sizes $A_{b}=1$ for controls, the event sizes for the topographical designs richly varied from $A_{b}≳0.1$ to $A_{b}≃1$.

A comparison of the distribution of bursting sizes in the different configurations is provided in the boxplots of Figure 1D. Data incorporate different experimental repetitions for the same configuration and compare young (DIV 7) and mature (DIV 14) cultures. For young cultures, whereas controls produced a narrow distribution with $\left\langle A_{b}\right\rangle ≃1$, tracks and squares were significantly shifted toward small values of $A_{b}$, with $\left\langle A_{b}\right\rangle ≃0.3$ and 0.35, respectively (p−values≪0.001 for control vs. tracks/squares). On maturation, the distribution of bursting sizes remained high for controls, indicating that these cultures activate in a coherent manner in all its lifespan. For tracks and squares, the distributions shifted toward higher $A_{b}$ values (more prominently for squares), with $\left\langle A_{b}\right\rangle ≃0.4$ and 0.6, respectively, but the $A_{b}$ distributions were still significantly different than controls (p−values≪0.001). We hypothesize that this increase in busting sizes upon maturation is associated with either an overall stronger interconnectivity in the network, longer average axons, or both. These connectivity changes smoothed out the impact of topography on dynamics and favored a higher presence of network-wide bursts. The validity of this hypothesis is discussed later in the context of effective connectivity analysis. On the other hand, we also observed that the $A_{b}$ distributions for tracks and squares were also significantly different ($p=7.5\times 10^{-6}$ at DIV 7 and p≪0.001 at DIV 14), indicating that their repertoire of dynamic states was modulated by the specific PDMS pattern at play.

The variety in activity patterns, which is reflected both in the structure of the raster plots and the distribution of $A_{b}$ values, can be quantified through a single parameter termed *dynamical richness* Θ.^22^which varies between Θ=0 for perfectly coherent or random activity and Θ=1 for maximally patterned activity, i.e., with all possible neuronal coactivation patterns present, from few neurons to the entire network. Figure 1E shows the results for the evolution of Θ as a function of DIV for the three configurations, averaged out among different repetitions. Although Θ≲0.1 for controls, with small changes along development, Θ exhibits at short DIV a much larger Θ≃0.33 and Θ≃0.25 for tracks and squares, respectively, remaining high for about a week until it decreases after DIV 14 as network-wide bursting in the topographical designs strengthens, i.e., $A_{b}$ distributions shift toward higher values (Figure 1D).

### Topographical cultures give rise to a rich repertoire of spatiotemporal activity patterns

The burst size $A_{b}$ captures the fraction of the network that activates coherently in a short time window but does not inform about the spatiotemporal structure of a burst. To explore this aspect, Figure 2A provides image plots of representative bursts’ evolution across the culture for the different configurations. For tracks and squares, we included data for young (DIV 7) and mature (DIV 14) cultures because they changed in dynamic behavior along maturation. This was not the case for control cultures, which exhibited whole-network bursts in all their evolution, and therefore only data at DIV 7 is shown. In the image plots we used a black-yellow color scheme to portray the activation time of each ROI, and marked the initiation point of the burst spatiotemporal front as a white circle. Regions of the culture with no activity are shown in gray.

For controls, activity encompassed the entire culture in all bursts and propagated as a quasi-circular front, which is reflected by a progressive change in color (from black to yellow) for ROIs gradually further from the origin of activity. Activity propagation was fast, with the bursts crossing the 6 mm diameter of the culture in about 0.2−0.3 s. Bursts also started approximately in the same location. Thus, the whole-network activation and the similar location of burst initiation shaped altogether a very rigid system. In the tracks configuration, by contrast, different sizes and propagation schemes were present (Videos S3 and S5), in combination with a richer variability in initiation points. Indeed, at DIV 7, some bursts encompassed just a couple of tracks (front #36), the entire culture (#42) or half of it (#53). Propagating fronts required about 3−4 s to propagate over the culture, *i*.*e*., bursts were an order of magnitude slower than in controls. As the culture matured, the bursts maintained this variability in sizes and initiation points, although there was a tendency for the sizes to encompass larger areas. Bursts extending only one or two tracks were rare in these mature networks. For squares, we observed that young cultures exhibited a rich variability in burst structures (Video S4), which encompassed either specific regions of the culture (bursts #1 and #148) or its entireness (#29). Burst propagation took about 1−2 s to cross the system, i.e., in between controls and tracks. The most prominent characteristic of the squares configuration, however, is that for mature cultures at DIV 14 there was a tendency for the bursts to cover large areas. Fragmented activity was rare (bursts #7 and 45) and most of bursts filled the entire culture (#19).

Video S5. 20 min of spontaneous activity in a full 6 mm diameter cultures with ‘tracks’ topography at DIV 14,related to Figures 1, 2, 4 and S3 Spontaneous activity for a ‘tracks’ culture at DIV 14. The video corresponds to the same culture as in SV3 but played at 40x speed to cover 20 min of actual recording. This allows to better appreciate the variability in activity patterns and how these patterns shape unique functional communities.

In Figure 2B we provide a diagram that compares in a compact manner the spatiotemporal structure of all bursts for each configuration and day *in vitro*. As explained in Transparent Methods, each color band in the diagram represents a burst. The width of the band indicates the number of participating neurons in each burst, whereas the color scale itself portrays the spatiotemporal evolution. Conceptually, those bursts that propagate similarly share the same color structure. The bursts are ordered in the yaxis according to a similarity analysis that identifies groups of akin bursts. Similarity was based on Pearson’s correlation among all pairs of bursts in combination with community detection (Figure 2C, white boxes). For Figure 2B, the groups of similar bursts are separated by a black line, and the gray discs with a number show the id of the bursts portrayed in Figure 2A. For controls, all bursts practically comprised the whole network and therefore they fill the width of the diagram. In addition, most of the color bands evolve from dark blue to clear blue, indicating a similar activity propagation across the culture. For tracks at DIV 7 three distinct groups appeared in the diagram and were associated with activity extending a couple of tracks (top group), most of the culture (central group) or half of it (bottom group). The color scheme of the bursts was richer than in controls, indicating that spatiotemporal propagation was more varied. These three distinct groups were preserved upon maturation, although the groups were more similar among themselves and color schemes were more uniform. A similar trend was observed in the squares configuration. Three distinct groups of bursts were clear at DIV 7, which correspond to typically small yet compact areas of the culture (top), quasi full-culture activations (center), and small activations in scattered areas (bottom). These groups practically vanished at DIV 14 as most of the bursting events comprised the entire culture.

### Immunostaining reveals connectivity traits induced by the PMDS topography

**Figure 3:**
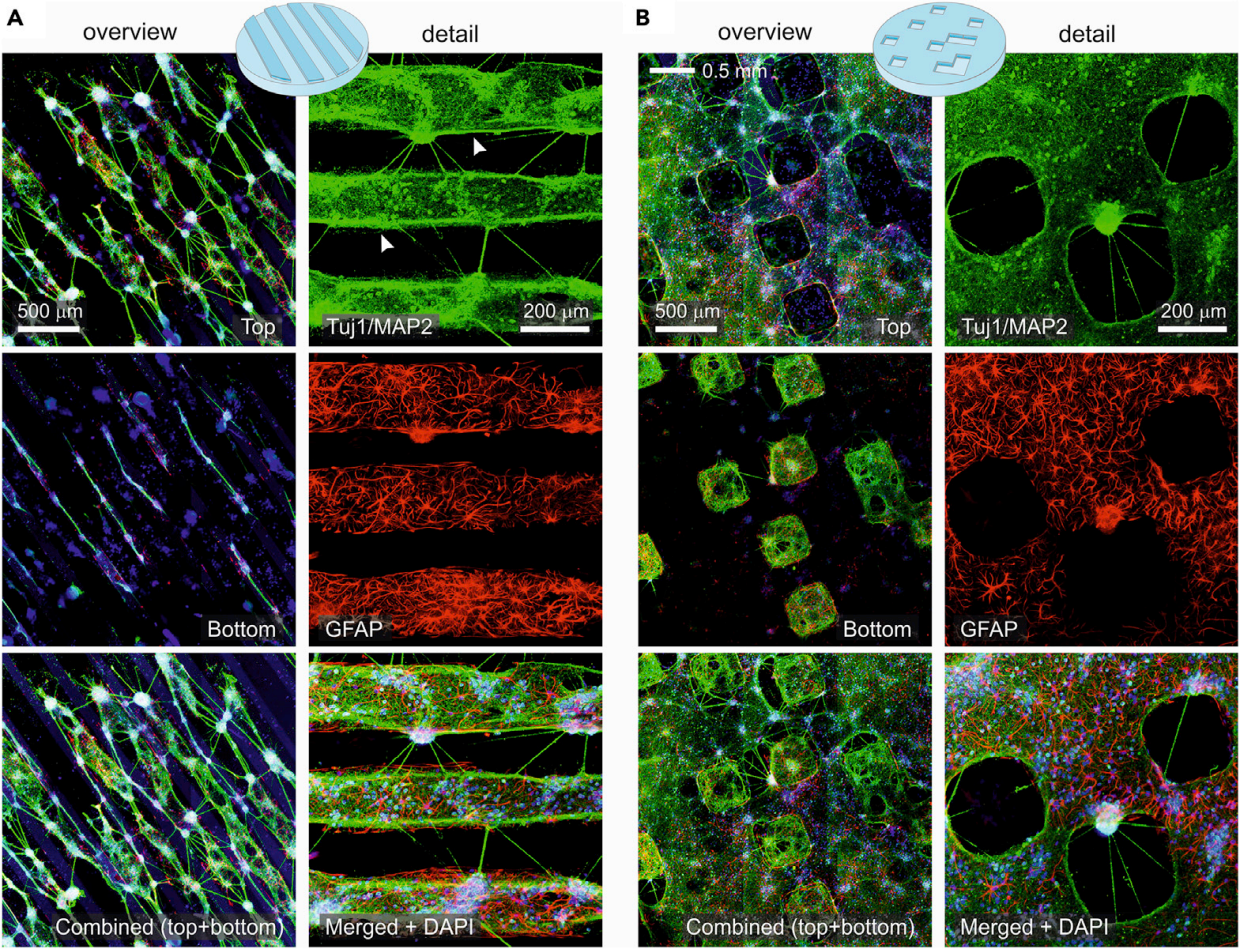
Anisotropic connectivity in PDMS topographical cultures revealed by immunostaining (A) Representative immunohistochemical images of neurons grown on PDMS topographical tracks at DIV 14, providing a broad overview (left) and a detail (right). For the overview, images show neuronal processes in green, astrocytes in red and cell nuclei in blue, with focus at the top part of the topography, the bottom part, and a combination of them. For the detail, images correspond to the top part of the design only and depict neuronal process (green), astrocytes (red), and the combination of these channels with cell nuclei (blue). Neuronal connections are more abundant in the direction of the tracks than transverse to them. White arrows mark neuronal processes that follow the edge of the topographical design. (B) Corresponding images for the squares configuration at DIV 14. Connectivity is abundant at the bottom part of the square designs, shaping small microcircuits by themselves that interconnect with the top part.

To understand the origin of the rich repertoire of activity patterns, we carried out an immunohistochemical analysis on the tracks and squares configurations. As shown in Figure 3, we were interested in identifying neuronal processes (green), astrocytes (red) and cell nuclei (blue). For tracks (Figure 3A), confocal images covering a field of view on the order of mm (‘overview’, left) revealed that neuronal processes extended preferentially along the direction of the tracks, both at the top and at the bottom of the PDMS relief, and that connectivity in the transverse direction was by comparison very minor (see also Video S1). Immunostaining also revealed that neuronal processes often tended to follow the edges of the relief, particularly at the top of the design (Figure 3A, detail, white arrowheads). Thus, topography provided guidance to connections, which were funneled along the tracks and shaped a highly anisotropic connectivity. A detail of the images (Figure 3A, right) allows us to clearly visualize the difference in connectivity along and across tracks. This difference provided the seed for shaping track-oriented, weakly coupled microcircuits that ultimately rendered rich spatiotemporal patterns. The detail immunostaining images also reveal the abundant and uniform distribution of astrocytes in the cultures, which contrasts with the aggregation of cell nuclei (Figure 3A, bottom), a trait that could also help to enrich connectivity microcircuits and varied emerging dynamics.

The immunohistochemical analysis for the squares configuration is provided in Figure 3B, both at the mm scale (left) and in the detail (right). For this configuration, the relief favored the formation of strongly connected islands at the bottom part of the relief which, in turn, connected with other islands or with the top part of the PDMS. Astrocytes and neurons were also abundant, although the latter were more homogeneously distributed as compared to tracks. Thus, for squares, the presence of spontaneous activity as ‘patches’ in Figure 2A (squares, DIV7) is most likely because of the activation of relatively isolated groups of neurons either at the top or at the bottom of the topographical relief. The interaction among the top and bottom parts, however, is complex because the entire network sporadically activated in a coherent manner. We note that, as in tracks, the relief facilitates the formation of interacting distinctive microcircuits that shape a rich variety of spontaneous activity patterns. These microcircuits are not stable in time, but rather continuously evolve. Indeed, the observation that activity at DIV 14 mostly encompasses the entire network indicates that there is global tendency for the microcircuits to gradually blend together and cast a much more uniform overall connectivity that translates into whole-network bursting events.

### PDMS topography impacts on burst initiation and velocity of burst propagation

**Figure 4:**
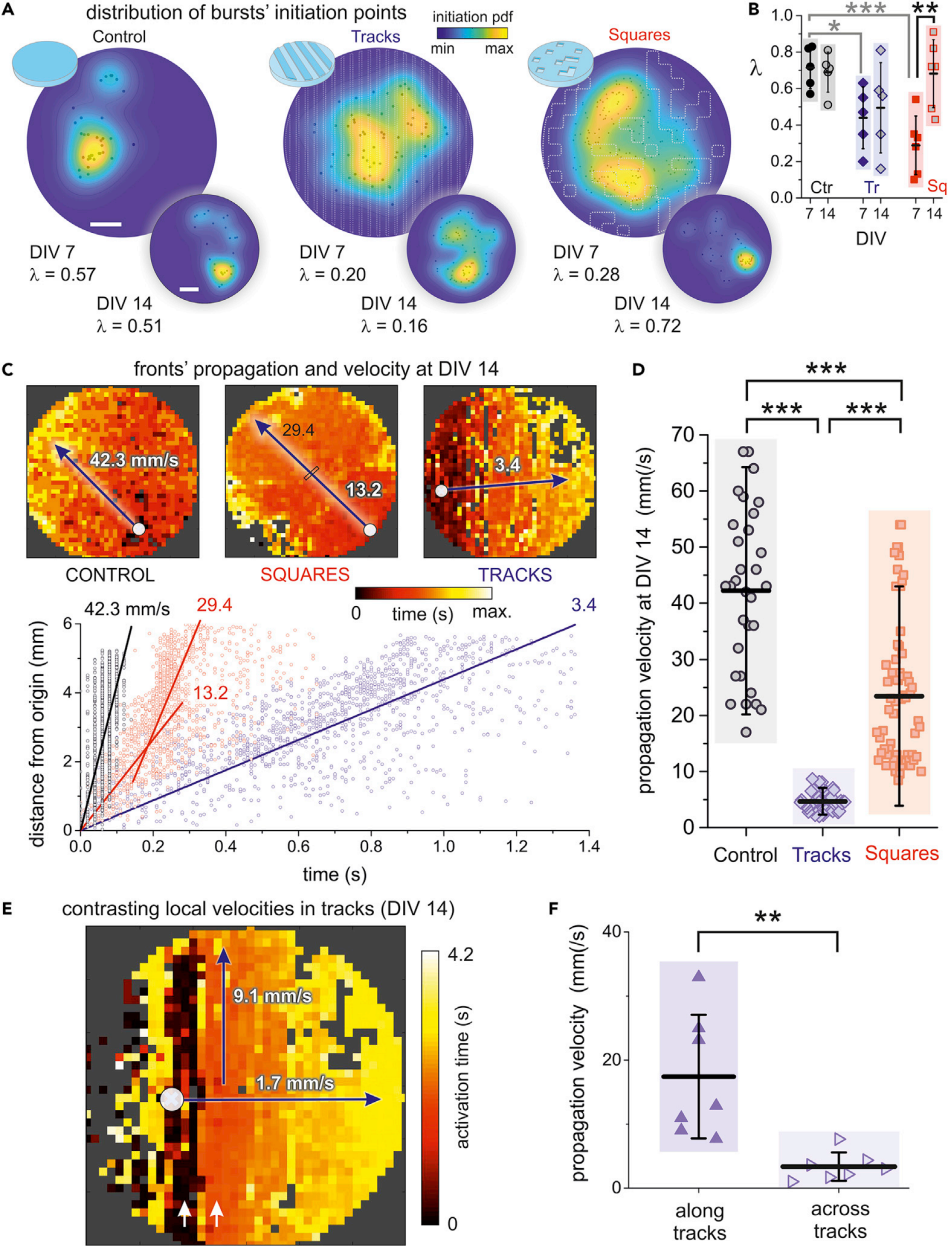
Burst initiation and velocity of propagation bursts in PDMS topographical cultures (A) Spatial distribution of burst initiation points (black dots) and probability density functions of burst initiation (pdf, blue-yellow colormap) for the PDMS configurations shown in Figure 2 and comparing two days *in vitro*, DIV 7 and DIV 14. λ is the Gini coefficient and indicates the degree of focalization of burst initiation. White scale bars are 1 mm and apply to all culture types. (B) Boxplots of the distribution of λ values for all experimental repetitions. At DIV 7, the λ distribution for controls is significantly higher (gray asterisks) than for tracks/squares. The λ distribution for squares at DIV 14 is significantly higher than the one at DIV 7 (black asterisks). ∗p < 0.05, ∗∗p < 0.01, ∗∗∗p < 0.001 (Student’s *t-*test). (C) Top, representative network bursts at DIV 14 that encompass the entire network and whose propagation is compatible with a circular or flat front. The white dot marks the origin of activity. Bottom, corresponding determination of velocity propagation as linear fits, where the Euclidean distance of each ROI to the origin of activity is plotted as a function of its activation time. The squares configuration exhibits a sudden, 2-fold increase in velocity. All linear fits have Pearson’s correlation coefficients r≳0.95. (D) Boxplots of propagation velocities for the three configurations, showing that fronts in tracks or squares configurations propagate much slowly than on controls. ∗∗∗p < 0.001 (Student’s *t-*test). (E) Local velocities in the tracks configuration, illustrating that activity propagation along the tracks (vertical direction) is much higher than across them (horizontal direction). Arrows marks tracks with high propagation speed and therefore very small color variation. (F) Boxplots of velocity propagation for different fronts of the same culture. On average, propagation along tracks is 18 mm/s, about 6 times larger than across tracks, which is of 3.5 mm/s ∗∗p < 0.01 (Student’s *t-*test).

Figure 4A provides the spatial distribution of burst initiation for the configurations shown in Figure 2 at two stages of culture maturation (DIV 7 and DIV14). In the panels, the black dots represent the spatial location of each observed burst whereas the blue-yellow colormap shows the corresponding probability distribution function of burst initiation. The degree of activity focalization, *i*.*e*., the tendency for spontaneous activity to initiate in the same location, is quantified through the Gini coefficient λ, which is 0 for a spatially equiprobable initiation, and 1 for a point-concentrated initiation occurrence. For controls (left), most of the bursts at DIV 7 started in the same neighborhood, leading to highly focalized distribution function (yellow spot) with λ≃0.57. This focalization was maintained at DIV 14 (λ≃0.51), although the location of the most probable initiation points varied because of global connectivity changes during maturation. By contrast, activity initiation for the tracks configuration was substantially more extended at DIV 7 (λ≃0.20), a trait that was maintained upon maturation (λ≃0.16 at DIV 14). Hence, topographical tracks not only help shaping connectivity anisotropies that enriched spontaneous activity but that these anisotropies were maintained upon maturation. For squares, initiation was spatially extended at DIV 7 (λ≃0.28) but became focalized upon maturation (λ≃0.72 at DIV 14). This focalization is consistent with the observed whole-network bursting and the gradual loss of connectivity anisotropies upon maturation.

We extended the above analysis to all culture types and repetitions. As shown in Figure 4B, the overall distribution of λ values for controls at DIV 7 was significantly higher than in tracks or squares (gray asterisks, p=0.017 and $p=8.0\times 10^{-4}$ respectively), indicating that activity initiation foci in patterned cultures were consistently more varied. At DIV 14, the λ distributions were not significantly different between controls and tracks/squares, indicating a trend toward a more concentrated activity initiation in all configurations. We note, however, that the dispersion of λ values for tracks/squares was important, and that it accentuated from DIV 7 to 14, with broader λ distributions for the latter day. Of interest, the tracks configuration maintained an average λ that was similar at DIV 7 and 14, suggesting that the dynamic alterations caused by the tracks topography were preserved. For squares, there was a significant increase in overall λ values from DIV 7 to 14 (p=0.0029), and with an average λ comparable to the one of controls.

We also investigated the velocity of propagating fronts in detail. We considered data at DIV 14 because most fronts encompassed large areas of the 6 mm culture, which provided sufficient statistics for a reliable analysis. The top panels of Figure 4C show representative spatiotemporal fronts for the three configurations, which evolve as quasi-circular fronts from the origin of activity (white circle). As shown in the bottom graph of Figure 4C, their characteristic propagation velocity was obtained by linear regression of d(t) data (solid lines), where d is the distance of each ROI to the origin of activity and t its activation time. The measured velocity was v≃40 mm/s for controls and substantially decayed to v≃13 and 3 mm/s for squares and tracks, respectively. Pearson’s regression coefficients in all three cases were r≳0.95. Despite the goodness of the linear regression approach there was, however, a noticeable dispersion in the data for squares and tracks that indicates strong changes in the propagation of the fronts at local scales. For squares, for instance, one could identify a first regime (in the range 0−0.2 s) of slow propagation with v≃13 mm/s followed by a second one (0.2−0.3 s) of fast propagation with v≃30 mm/s, which suggests an abrupt change of the underpinned local connectivity during front evolution. For tracks, an inspection of the data revealed that the strong dispersion of some points, with identical time activation of ROIs that were ≃6 mm apart, was associated with a much faster propagation of activity along the tracks than transverse to them, as discussed below. The analysis of the velocity using linear regression was consistent across experimental realizations (Figure 4D), with significantly different velocities between controls (⟨v⟩≃42.2±14.7 mm/s), tracks (4.7±1.6 mm/s) and squares (23.4±13.0 mm/s).

The contrast in propagating velocities along PDMS tracks or transversally to them outlined above is analyzed in more detail in Figures 4E and 4F (see also Video S3). For the illustrative spatiotemporal front of Figure 4E we observed that the color scheme along tracks (vertical axis of the image plot) was practically uniform, with characteristic black and red bands (white arrows) that revealed the fast activation of the whole track, typically in a time window on order of 0.3 s. Conversely, the color variation transverse to tracks (horizontal axis) smoothy varied from black at the origin of activity to yellow at the right edge of the culture, leading to a propagation time of 4.3 s. The corresponding velocities were about an order of magnitude dissimilar, with $v_{∥}≃9.1$ mm/s along tracks and $v_{⊥}≃1.7$ mm/s transversally to them. This dissimilarity was preserved across experimental repetitions and was significantly different (Figure 4F, p = 0.0027), and on average we obtained $\left\langle v_{∥}\right\rangle =17.4\;\pm \;9.6$ mm/s and $\left\langle v_{⊥}\right\rangle ≃3.3\;\pm 2.2$ mm/s.

### Effective connectivity analysis identifies unique network organization traits in topographical cultures

**Figure 5:**
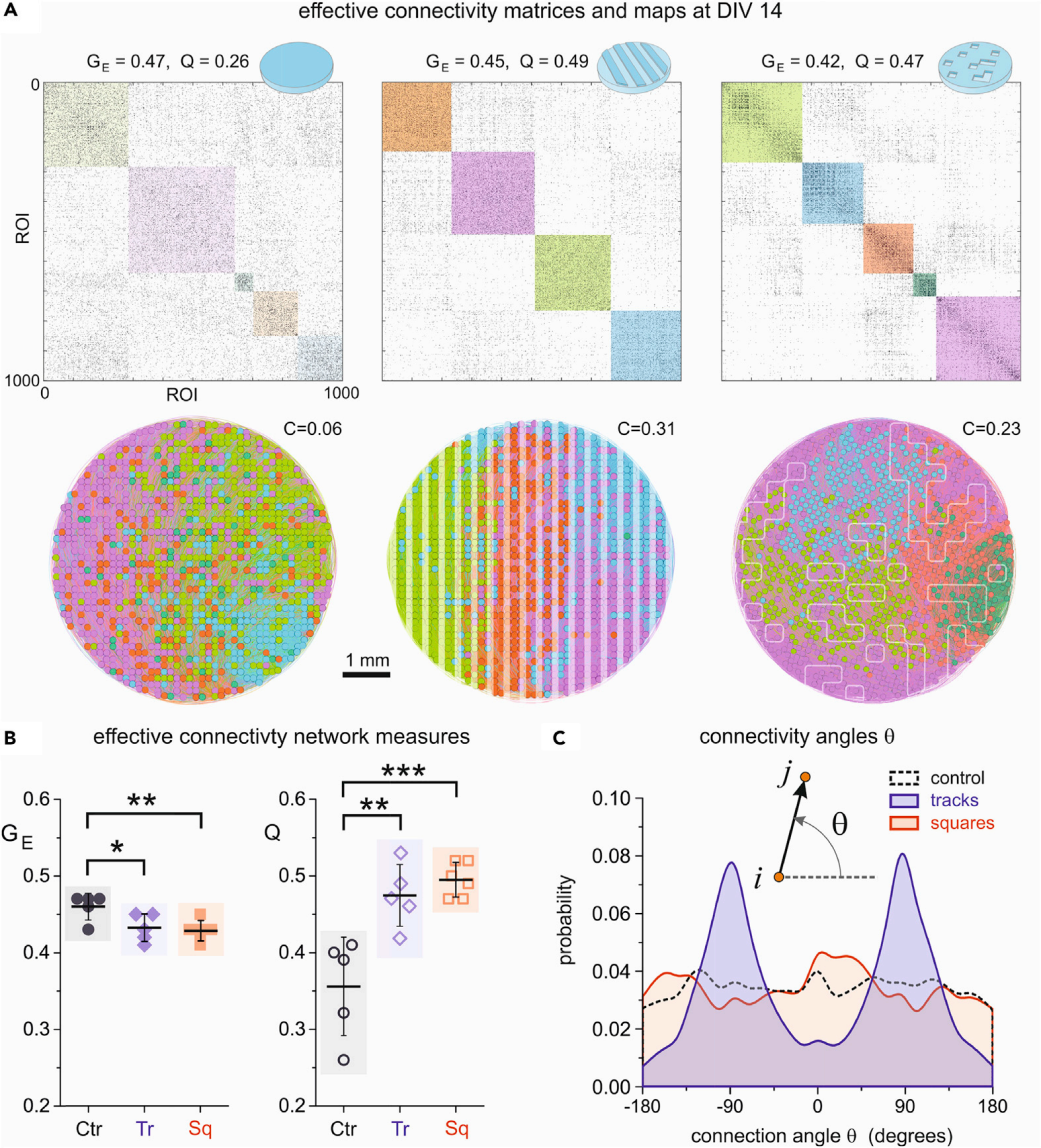
Effective connectivity analysis in PDMS topographical cultures (A) Top, adjacency matrices of effective connectivity among ROI pairs for the three PDMS configurations at DIV 14. The global efficiency $G_{E}$ and modularity Q values of the networks are indicated on the top. Color boxes along the diagonal of the matrices highlight functional modules, with their color intensity proportional to their strength. Bottom, corresponding network maps, where each dot is an ROI color coded according to the functional module it belongs to. The value of C accompanying each map indicates the average spatial compactness of the functional modules, which is markedly high for tracks. (B) Boxplots of comparing the network measures $G_{E}$ and Q for the three configurations and including all experimental repetitions. Flat PDMS cultures exhibit a significantly higher $G_{E}$ and lower Q than the other configurations. ∗p < 0.05, ∗∗p < 0.01, ∗∗∗p < 0.001 (Student’s *t-*test). (C) Distribution of connectivity angles θ, where θ is the angle formed between two effectively connected neurons i→j and the horizontal axis. The distributions for both controls and squares are approximately flat, whereas the one for tracks is markedly peaked at 90 and −90°, indicating strong neuronal communication along the tracks.

We used generalized transfer entropy (GTE) to infer causal relations among ROIs in the neuronal cultures (Figure 5), and from them extracted network measures that exposed functional organizational traits. In all cases we used the data presented in the previous figures and at DIV 14. Conceptually, the nodes of the computed effective networks are the ROIs in our experiments, whereas the links are the flow of information among those ROIs. The network traits that we explored include the Global efficiency $G_{E}$ and the modularity index Q. The former captures the capacity of the network to share information as a whole, whereas the latter informs about the existence of functional modules, *i*.*e*., groups of ROIs that tend to communicate within their group more strongly than with other groups in the network. Figure 5A, top, shows the obtained effective connectivity matrices for the three PDMS configurations, with the modules highlighted as color boxes along the diagonal. In all configurations we observed an abundance of effective connections both within modules and between them. All networks indeed exhibited a similar $G_{E}≃0.45$, indicating that neurons in the three configurations easily exchanged information globally. This is understandable in the context of the observed dynamics, in which whole-network correlated activity was present in the three cultures and thus global neuronal communication. The modularity Q, however, was clearly different among configurations, with Q≃0.26 for controls, Q≃0.49 for tracks and Q≃0.47 for squares. The low Q for controls indicates that ROIs within a module connected similarly among themselves and the rest of the network, *i*.*e*., the network effectually operated as a unique system. The high Q for the topographical designs indicates the presence of functional microcircuits, *i*.*e*., strongly interconnected neuronal assemblies.

The network maps associated with the connectivity matrices (Figure 5A, bottom) provide an additional insight into the impact of PDMS topography on neuronal network communication. These maps show the spatial location of the ROIs in the culture as circles, and color coded according to the functional module they belong to. Effective connections are present, but their abundance masks their individual identification. For controls, the functional modules are spatially interwoven over the area of the culture, a feature that is quantified by the average compactness C of the spots and that provided C≃0. This mixture of the modules indicates that a neuron tended to interact similarly with any other in the culture and at very long distances. By contrast, for the tracks configuration we observed that functional modules shaped compact spots in the culture (C≃0.31) that were aligned with the tracks themselves, indicating that the topographical relief orchestrated the functional organization of the culture. The spatial extent of the functional modules could be related to specific spatiotemporal activity patterns (Figure S3 and Video S5), which indicates a relation between network dynamics and functional traits. A similar compact functional organization was observed for the squares configuration (C≃0.23), although the shape of the modules did not concord with the arrangement of the PDMS design possibly because of the high connectivity between top and bottom parts at DIV 14.

The obtained results for functional organization in terms of $G_{E}$ and Q were maintained across experimental realizations (Figure 5B). $G_{E}$ was not significantly different for the three configurations, but Q for tracks and squares was significantly higher than for controls.

To complete the analysis, we investigated in more detail the emergence of local functional features in the topographical cultures. Given the capacity of tracks to funnel neuronal connectivity along their length, we first examined whether such a privileged direction was captured by the GTE effective connectivity analysis. For that, we computed the angle of effective connections relative to the horizontal axis and plotted the distribution of angles. As shown in Figure 5C, controls (black curve) showed a homogeneous distribution of angles that indicated an isotropic effective connectivity. A similar result was observed for the squares configuration (red), although the distribution exhibited strong fluctuations. For tracks (blue), however, neat peaks appeared at −90 and 90°, distinctly revealing a preferred vertical direction in neuronal communication. Thus, the effective connectivity analysis shown here demonstrated to be an invaluable tool to complete the activity and immunostaining analyses, and brought to light additional evidence that the PDMS relief help dictating the way in which neurons wired and communicated.

The observation that effective connections were primarily oriented along the tracks themselves is interesting and made us inquire whether we could also observe differences in the distribution of effective connectivity distances, defined as the Euclidean separation between any pair of effectively connected ROIs. Because effective connectivity reflects dynamics or neuronal communication, in principle these distances could extend the entire culture diameter of ∅ = 6 mm. For sake of discussion, we established that those effective connections that projected beyond 75% of the culture’s diameter were considered ‘long-range’, which corresponds to 4.5 mm. The analysis of the distribution of these distances for DIV 7 and DIV 14 is provided in Figure S4, with the data for DIV 14 corresponding to the networks of Figure 5A. The results show that, for controls, long-range effective connections were abundant both at DIV 7 and DIV 14, which is compatible with the observed network-wide bursting in all their developmental stages, whereas for tracks/squares long-range connections were rare at DIV 7 and substantially increased at DIV 14.

The increase in long-range effective connectivity upon maturation suggests that the induced PDMS anisotropy somehow blocked the capacity for fast whole-network communication at early stages of development. However, the impact of anisotropies was less prominent at later stages, possibly because of the growth of axons, which effectually integrated the entire network. To provide evidence for this idea, we carried out numerical simulations in which we used biologically realistic models of the three experimental culture types to, then, explore the emerging dynamics and effective connectivity as the average axonal lengths grew. An example of the constructed networks is provided in Figure S5A, and the results for the distribution of connectivity distances are provided in Figure S5B. The results show that longer axons clearly favored network-wide bursting and substantially increased the presence of long-range effective connections. Thus, we conclude that young cultures most likely exhibited short axons which, in combination with the anisotropy induced by PDMS patterning, confines activity in small neighborhoods, with rare network-wide bursting. Mature cultures, with long axons, favored global connectivity that smoothed out the underlying anisotropy and facilitated network-wide bursting. Numerical simulations also evince that effective and structural connectivity are related, but that one cannot freely take the former as a proxy for the latter.

**Figure 6:**
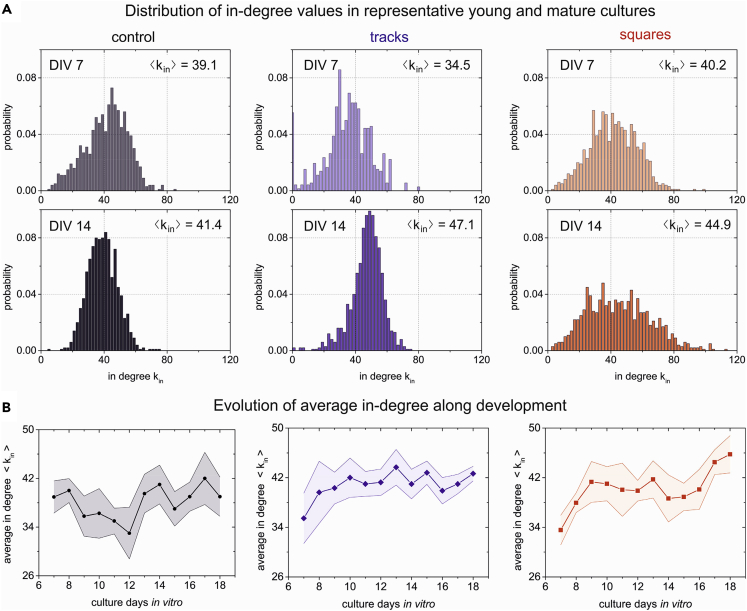
In-degree connectivity distributions and evolution of average connectivity along development (A) In-degree probability distribution functions for representative young (DIV 7) and mature (DIV 14) cultures, comparing controls with tracks and squares topographical cultures. The data at DIV 14 correspond to the matrices and maps provided in Figure 5. The value of $\left\langle k_{in}\right\rangle$ shown on the top-left corner of each distribution provides the average connectivity of the network. $\left\langle k_{in}\right\rangle$ is similar between DIV 7 and 14 for controls but increases by 36 and 12% (relative to the values at DIV 7) for tracks and squares, respectively. (B) Evolution of $\left\langle k_{in}\right\rangle$ for the three culture types along DIV and including all explored experimental repetitions (n = 5 for controls, 5 for tracks, and 6 for squares). Data are represented as mean ± SD of the mean. For controls, $\left\langle k_{in}\right\rangle$ oscillates around 40 connections/ROI along DIV, whereas for tracks and squares a clear growing trend in average connectivity is observed from DIV 7 to DIV 9–10, when connectivity appears to stabilize and later fluctuate.

Given the interesting insight that effective connectivity provides, we next investigated the local topological properties of the obtained effective networks, and considered the distribution of in-degrees, clustering coefficients and other properties. We initially focused on the analysis of in-degrees $k_{in}$ (incoming connections to a given ROI), comparing the shape of $k_{in}$ distributions between DIV 7 and 14 as well as the evolution of the average degree $\left\langle k_{in}\right\rangle$ along development. We considered the in-degree only because in a previous numerical study^30^ we showed that $k_{in}$ better captured the differences between neuronal networks grown in environments with strong spatial constraints. As shown in Figure 6A, $\left\langle k_{in}\right\rangle$ for controls did not substantially vary which, in the context of the observed persistent network-wide bursting of these cultures because the very beginning, indicates that the network maintains a similar functional behavior along development. Of interest, very small in-degrees are not present at DIV 14, which hints at an overall stronger connectivity caused by maturation, i.e., the realization of an overall well-connected network. For tracks, a change is noticeable, with an abundance of small in-degree values at DIV 7 that disappear at DIV 14. $\left\langle k_{in}\right\rangle$ grows from 34 (DIV 7) to 47 (DIV 14), a 36% relative increase. For squares, an increase in overall connectivity is also observed, from about 40 to 45, a 12% relative increase, much smaller than tracks, which is possibly because of the fact that the PDMS square patterns do not completely dominate the surface of the culture, i.e., substantial areas connect similarly as in a flat, control culture. However, the distribution of degree values for squares at DIV 14 is much broader than for controls, indicating that connectivity on the surface of the squares configuration may abruptly change. We believe that this broadness in $k_{in}$ values is related to the abrupt changes in the propagation velocity of fronts shown in Figure 4C.

The evolution of $\left\langle k_{in}\right\rangle$ along DIV for the three configurations is shown in Figure 6B. For controls, $\left\langle k_{in}\right\rangle$ oscillates around $\left\langle k_{in}\right\rangle ≃40$ and no clear developmental trend can be observed. Again, we think that this is because of the dynamical locking of the system in network-wide bursting. For tracks and squares, however, a clear development is observed, changing for both configurations from $\left\langle k_{in}\right\rangle ≃34$ at DIV 7 to $\left\langle k_{in}\right\rangle ≃42$ after DIV 10, about 25% relative increase. This development of effective connections is consistent with a dynamics (and therefore neuronal communication) involving small regions of the culture at DIV 7 to, later, encompass its full entireness. We note that fluctuations in $\left\langle k_{in}\right\rangle$ values were strong, as revealed by the relatively large error bars, indicating that cultures prepared in similar conditions easily follow different developmental paths.

We finally inspected topological properties of the networks at DIV 14 that were related with the functional communication and organization at local scales. They include the distribution of clustering coefficients, local efficiencies and betweenness centralities, averaged over experimental repetitions. The results are provided in Figure S6 and complete the global analyses ($G_{E}$ and Q) provided in Figure 5. In general, tracks and squares exhibited higher clustering coefficients and local efficiencies than controls, indicating a higher tendency for the topographical patterns to shape local subgraphs and functional communities, which in turn are related to the observed capacity of neurons to activate in small groups and not at unison as in controls. The betweenness centrality exhibited similar average values for the three configurations, although the distributions were strongly peaked toward zero except for tracks, possibly indicating that this configuration favored hubs formation, e.g., neurons connecting tracks transversally. We also included for completeness the averaged $k_{in}$ distributions among repetitions, leading to results that were consistent with those provided in Figure 6, particularly the broadness of $k_{in}$ values for squares. Altogether, these results show that there are differences in local network properties between culture types that are consistent with the overall results on dynamics, although these differences are not significant in statistical terms, an aspect that we elaborate in the discussion section.

## Discussion

Primary neuronal cultures are one of the most celebrated techniques in several multidisciplinary research fields, including physics of complex systems, neuroengineering and medicine. Their versatility, accessibility and ease of manipulation have made them ideal to investigate in a controlled manner phenomena as diverse as self-organization,^26^^,^^27^^,^^28^ repertoire of activity patterns,^22^^,^^31^ structure-to-function relationship,^12^^,^^14^^,^^29^resilience to perturbations^32^^,^^33^ and alteration upon disease.^34^^,^^35^ However, primary cultures grown on flat surfaces typically exhibit a strong bursting behavior in which all neurons activate together in a short time window and remain practically silent in between bursts. In the present study we showed that this all-or-none rigid behavior can be relaxed by incorporating spatial anisotropies on the substrate, in the form of topographical reliefs, that substantially enrich the repertoire of activity patterns and approach them to what is observed *in vivo*, where activations of different spatiotemporal structure coexist.

The spatial constraints promoted by the PDMS reliefs favored local connectivity and facilitated activity at a microcircuit level, but without suppressing whole-network dynamics. Thus, our design promoted the emergence of neuronal networks with balanced integration and segregation, *i*.*e*., where activity patterns comprising small regions of the culture coexisted with network-wide bursting (Figure 2). In other words, for a given patterned culture, both local computation and whole-network communication coexisted. Other studies investigated the realization of this integration-segregation balance by using modular designs,^20^^,^^22^^,^^36^ in which neurons were spatially confined in small areas. Our PDMS topographical modulation provides an alternative approach to such an *ad hoc* confinement, shaping dynamically rich networks in both space and time without fine-tuning the position of neurons and axons. We note that a broader modulation of the dynamical repertoire in our cultures could be achieved by altering the ratio between excitatory of inhibitory neurons or by decreasing the strength of excitatory synapses. All our experiments were conducted with both excitation and inhibition active. Given the role of inhibition in modulating activity,^37^^,^^38^ we argue that the repertoire of activity patterns could additionally be tuned by blocking GABA receptors in inhibitory neurons, which would make bursting events more similar among themselves, or by reducing the amount of excitatory transmission by blocking AMPA-glutamate receptors. The latter was explored for instance in modular networks,^22^ observing that the integration-segregation balance shifted toward higher segregation as excitation was reduced. The importance of inhibition in modulating the repertoire of activity patterns will be explored in future work, both *in vitro* and *in silico*.

We found a PDMS height of around 70 μm to be optimal, because it promoted a coarse axonal positioning and orientation in a neuronal neighborhood, *e*.*g*., along a PDMS track while allowing for easy interconnection with other neighborhoods. Our observation that connections follow the track ridges is in agreement with other studies that investigated in detail the impact of geometrical cues on axonal growth.^39^ PDMS heights of 100 μm or higher in our experiments often shaped isolated regions in the culture, whereas heights of ∼30 μm did not cause sufficient structural alterations to markedly modify network dynamics. Other studies also used PDMS reliefs to control connectivity in neuronal circuits, most notably holes and pillars of characteristic scales in the range 10−100 μm,^40^^,^^41^ ultrasoft PDMS,^42^ or by combining fine-tuned neuroengineering and microfluidics.^14^^,^^19^^,^^43^^,^^44^^,^^45^ In these works, authors reported a richer repertoire of activity patterns or the suppression of extreme bursting. In the context of these studies, the relevance of our PDMS reliefs is that cells’ neurite development is coarsely guided rather than fully delineated, allowing the circuit to retain its self-organization potential. The effort of imprinting ‘mesoscale architecture’ while allowing self-organization is conceptually similar to the studies on neuronal cultures with spatial aggregation.^28^^,^^29^^,^^46^ Aggregation helped neurons to connect within their neighborhood but without hindering long-range connectivity, shaping networks with a richer dynamic behavior and more varied activity initiation, as in our case.

In our study we used effective connectivity, computed from spontaneous activity, to better understand communication across the neuronal cultures, and as a complementary analysis to the observed dynamical changes in the patterned networks. It is important to emphasize that effective connectivity reflects communication among neurons and not structural paths or synaptic efficiencies, and therefore a direct quantitative analysis of structural connectivity is not possible based solely on the analysis of the GTE-inferred effective data. We explored the difficulty of unraveling structural traits from effective connectivity by running simulations of the patterned networks and computing specific network descriptors for both the ground-truth topology (structure) and effective connectivity data. As illustrated in Figure S7, the distribution of structural clustering coefficients was similar among culture types even though the networks behave differently from a dynamical point of view. The effective clustering coefficients had some differences among configurations, but one could not easily infer structural traits from them. Thus, we hypothesize that to infer structural details from effective connectivity one needs, first, to combine as many descriptors as possible, e.g., global and local properties, connectivity distances and angles, hubness and connectivity cliques, and many others,^47^^,^^48^ and second, to combine recordings of spontaneous activity with electrical or optogenetic stimulation^49^^,^^50^ to unveil which neurons directly respond to a specific stimulus and gradually construct the network map of interactions.

The contrasting velocities of activity propagation in the tracks configuration, with measured velocities about 5 times larger along tracks than across them, can be put in context of theoretical models^51^^,^^52^ and experiments on activity propagation in one-dimensional neuronal cultures.^53^^,^^54^^,^^55^ These studies revealed that the velocity of activity fronts depended on average neuronal connectivity and synaptic strength. If we assume that synaptic strength is similar in all neurons in the culture, then we conclude that connectivity along tracks was 5-fold higher than transverse to it. This difference is consistent with the functional data of Figure 5C, in which effective connections along tracks were about 8 times more abundant than across tracks. We also remark that the maximum velocity that we measured along tracks was about 30 mm/s, which is slower compared to the data provided in the study of Feinerman et al.,^53^ who measured velocities in the range 40–80 mm/s. In Feinerman’s study, however, activity was solely driven by excitatory neurons, whereas in our networks both excitation and inhibition are active. Thus, the presence of inhibition, which effectually reduces average connectivity from a network dynamics perspective,^29^^,^^56^^,^^57^ is possibly the reason for the comparatively low velocities measured in our experiments.

Given the importance of spatial constraints in molding neuronal circuitry architecture and function,^58^ it is interesting as a future exploration to investigate the impact of different degrees of connectivity restrictions. Chemical patterning^22^^,^^45^ offers the delineation of precise circuits but with restricted self-organization capacity, whereas PDMS topographical modulation offers a broader flexibility at an expense of a poorer control on physical wiring. We observed in our experiments that different repetitions of the tracks or squares designs led to neuronal circuits with similar global dynamic behavior but with different functional details. For instance, the distribution of initiation points (Figure 4) and functional modules (Figure 5) varied across repetitions, indicating different mesoscopic evolution. In addition, some of the explored cultures tended to become more integrated and with increasingly stronger network-wide bursting as they matured, suggesting that the initially imprinted in homogeneities were erased at long term (Figure S6). This loss of richness was particularly strong in the squares configuration. We hypothesize that, to better approach brain-like behavior *in vitro*, an optimal experimental system would be one that combines topographical and chemical patterning, thus preserving key functional traits without the loss of flexible self-organization. We also conjecture that external stimulation, e.g., as in Poli et al.,^59^ may be a necessary ingredient to shape circuits with long-lasting functional features.

Our neuronal cultures were monitored using calcium imaging to detect neuronal activity at different heights of the PDMS patterns. Technologies based on planar electrodes such as MEAs were not suitable for our investigation because they require the neurons to be located on the surface of the MEAs chip. However, the concept of constraining neurons or connections to enrich the dynamics of neuronal cultures has been widely explored using planar MEAs,^15^^,^^17^ with the additional advantage that neurons could be locally stimulated. Successful concepts related to our work included modular designs,^20^ predefined connectivity through axonal guidance^60^ and interconnected subpopulations of neurons.^61^^,^^62^^,^^63^^,^^64^ Studies using modularity and guidance shaped networks that qualitatively resembled our tracks configuration, and authors reported a richer dynamic repertoire when modularity and guidance were present as compared to non-modular isotropic configurations. The studies using interconnected subpopulations of neurons showed that the dynamical and functional richness of the networks substantially increased when neurons from diverse origin (e.g., cortex or hippocampus) were wired together, overall shaping a true brain-on-chip system that not only captured functional complexity but also the intrinsic neuronal variability of the brain. In the context of our work, we hypothesize that the richness that we observed could be further increased by placing different neuronal types along groups of tracks or in the valleys of the squares configuration.

Finally, we note that our experiments may be of interest for those studies that use neuronal cultures as models for neurological disorders *in vitro*. These studies often explore the alterations in network collective activity caused by a disease. For instance, in a recent study of Parkinson *in vitro,*^65^ authors observed that the affected networks exhibited a much higher number of network-wide bursts as compared to healthy controls. Although their results were conclusive, the investigation was made difficult by the tendency of standard, glass-grown neuronal cultures to exhibit persistent whole-network bursting. Thus, we argue that the use of PDMS topographical substrates may help to prepare networks whose activity is much varied since early development, identify the impact of a disease in network formation, activity and functionality, as well as their evolution along time.

### Limitations of the study

Neurons in our experiments were plated in a homogeneous manner in PLL-coated PDMS surfaces. However, we often observed that neurons strongly aggregated after few days, or that some areas of the PDMS relief, generally at the bottom, were not occupied by cells. This problem was present both in the tracks and squares configurations, and often led to large empty areas for the latter. We ascribe this lack of homogeneity to the PLL coating, which possibly was not sufficiently uniform for neurons to adhere to the surface, or to capillary forces that caused the trapping of air bubbles and blocked coating. These inhomogeneities can be observed for instance in the fluorescence image of the tracks configuration in Figure 1. We note that fluctuations in local density accentuated the anisotropies induced by the relief and, in turn, amplified the variability of the spatiotemporal fronts. Specifically, for the tracks configuration, the distinct parallel and transverse velocities are possibly favored by the contrasting neuronal densities between top and bottom parts. Nonetheless, we used high-resolution phase contrast images and immunostaining to reject cultures in which neurons grew as isolated patches. All studied cultures here contained neurons that were globally interconnected and exhibited episodes of coordinated activity that encompassed from few neurons to the entire network.

On the other hand, in this study we were interested in the collective behavior of mm-sized cultures rather than in the precise individual dynamics of their constituting neurons. The need to access a large field of view in combination with limitations in image resolution imposed by the fluorescence camera, made not possible to resolve single cells. Hence, we analyzed network activity using an ROI approach. To investigate whether this approach could create artifacts, we run experiments in smaller, 4 mm diameter cultures in which both single neuron monitoring and ROIs could be used.^29^^,^^66^ Similar qualitatively results were obtained when comparing both approaches for all major dynamic and functional descriptors. Thus, the ROIs analysis can be viewed as a coarse-graining approach, which suffices to capture interesting mesoscale phenomena as far as the spatial extent of these phenomena is larger than the characteristic neuron size. In our case, network bursts and functional modules covered areas on the order of few mm, much larger than the 10μm diameter of a neuron. We believe that this coarse-graining may be a source of inspiration to explore neuronal circuits at different scales and may help bridging the gap between *in vitro* networks and naturally formed neuronal circuits. Related to this, it is important to emphasize that we considered rat primary cortical cultures for our experimental design. Alternative cells models such as human induced pluripotent stem cells (hiPSCs) may broaden the spectrum of dynamical and functional traits shown here. Indeed, hiPSCs cultures grown on flat substrates already exhibit a higher individual activity and a richer repertoire of coordinated activations,^65^^,^^67^ as opposed to rat primary cultures that show a strongly rigid bursting behavior.

## STAR★Methods

### Key resources table

**Table:** 

REAGENT or RESOURCE	SOURCE	IDENTIFIER
**Antibodies**		

Monoclonal Anti-β-Tubulin III antibody produced in mouse	Sigma-Aldrich	Cat. #T8660; AB_477590
Anti-MAP2 (2a+2b) antibody, Mouse monoclonal	Sigma-Aldrich	Cat. #M1406; AB_477171
Anti-Glial Fibrillary Acidic Protein (GFAP)−Cy3™ antibody, Mouse monoclonal	Sigma-Aldrich	Cat. #C9205; AB_476889

**Bacterial and virus strains**		

AAV9.Syn.GCaMP6s.WPRE.SV40	Addgene	Cat. # 100843-AAV9

**Experimental models: Organisms/strains**		

Sprague-Dawley rat	University of Barcelona	N/A

**Chemicals, peptides, and recombinant proteins**		

DAPI-fluoromount–G	Shouthern Biotech	Cat. # 0100–20
L-15 Medium	Thermo Fisher	Cat. # 11415–049
MEM Eagle’s Minimum Essential Medium	Thermo Fisher	Cat. # 21090–022
Glutamax	Fisher Scientific	Cat. # 35050–038
Glucose	Sigma-Aldrich	Cat. #G5400-1KG
Horse Serum	Thermo Fisher	Cat. # 26050088
Fetal bovine serum	Thermo Fisher	Cat. # 16140071
FUDR	Sigma-Aldrich	Cat. #F0503
B27	Fisher Scientific	Cat. # 17504044
Poly-L-Lysine	Sigma-Aldrich	Cat. #P4707
Boric Acid	Sigma-Aldrich	Cat. #B19334-500G
Borax Anhydrous	Sigma-Aldrich	Cat. # 71997–100G
PFA	Sigma-Aldrich	Cat. #P6148
PBS	Thermo-Fisher	Cat. # 10010023
Triton	Sigma	Cat. # T8787
Normal Donkey Serum	Jackson Immuno research	Cat. # 017-000-121

**Deposited data**		

Neuronal cultures on PDMS topographical patterns: experiments and simulations (https://doi.org/10.17632/8yp4xb6d3s.1)	Mendeley Data	https://data.mendeley.com/datasets/8yp4xb6d3s/1

**Software and algorithms**		

NETCAL: An interactive platform for large-scale, NETwork and population dynamics analysis of CALcium imaging recordings	Zenodo^34^^,^^68^	https://zenodo.org/record/1119026#.Y20nUXbMKUk
Gephi	Bastian et al., 2009^69^	https://gephi.org/
MATLAB 2018a	MathWorks	https://www.mathworks.com/
Origin 9.1	OriginLab	https://www.originlab.com/

**Other**		

PDMS kit: Sylgard 184	Ellsworth Adhesives	Cat. # 2137054

### Resource availability

#### Lead contact

Further information can be requested by the lead contact, (jordi.soriano@ub.edu).

#### Materials availability

This study did not generate new unique reagents.

### Experimental model and subject details

#### Primary rat neuronal cultures

In all experiments, primary neurons from Sprague-Dawley embryonic cortices at days 18–19 of development were used and following identical protocols as described previously.^29^^,^^57^^,^^66^ Rats were provided by the animal farm of the University of Barcelona. Animal manipulation and dissection of the embryonic cortices were carried out under ethical order B-RP-094/15–7125 of July 10^th^, 2015.

Cortices dissection and cell culturing were carried out at the laboratory of Dr. Soriano at the Faculty of Physics of the University of Barcelona. Briefly, dissection was carried out in ice-cold L-15 medium (Gibco), cortical tissue dissociated mechanically by repeated pipetting, and neurons suspended in plating medium [90% Eagle’s Minimum Essential Medium (MEM, Invitrogen) with 5% Horse Serum (HS, Invitrogen), 5% Bovine Calf Serum (Invitrogen) and 1 μL/mL B27 (Sigma)]. Prior culturing, PDMS surfaces were submerged overnight in the adhesive protein Poly–L–Lysine (PLL, Sigma-Aldrich) at a concentration of 10 mg/mL PLL in Borate Buffer. Neurons were plated on these surfaces and led to cultures with a neuronal density of about 400 neurons/mm^2^. Neurons were homogenously distributed over the surface, although some aggregation existed. One day after plating, cultures were infected with adeno-associated viruses bearing the GCamp6s calcium sensor under synapsin-I promoter (AAV9.Syn.GCaMP6s.WPRE.SV40, Addgene). Four days afterward, at day *in vitro* (DIV) 5, plating medium was replaced by changing medium (90% MEM, 10% HS and 0.5% FUDR) to limit glial growth. At DIV 8 the medium was switch again to final medium (90% MEM and 10% HS) and refreshed periodically every 3 days.

Only neurons expressed the calcium sensor under the CaMKII promoter. Thus, although cultures contained both neurons and glia, only neuronal activity was visualized.

4 wells (8 PDMS cultures) were prepared in each dissection and were kept in 4-well plates (Nunc) in which only two wells were used. This facilitated the consecutive recording of different cultures while minimizing possible alterations in those wells that were not recorded at that moment. Cultures were incubated at 37°C, 5% CO_2_, and 95% humidity. Spontaneous activity emerged by DIV 5, but GCAMP6s expression was not sufficiently strong for reliable imaging until DIV 7.

### Methods details

#### PDMS topographical reliefs

Topographical substrates were prepared by using a specially designed printed circuit board (2CI Circuitos Impresos, Spain) that served as a negative mold for the desired topographical design. As shown in Figure S1A, the printed circuit was form by two layers, a bottom one of uniform fiberglass 2 mm thick and a top one of cooper deposits 70 μm high that shaped different designs. This height of the copper was constant along the board. For the present work, two main designs were used and termed ‘tracks’ and ‘squares’ (Figure S1B). ‘Tracks’ consisted of parallel rectangular bands 300 μm wide and 20 mm long and separated by 300 μm. ‘Squares’ consisted of randomly positioned squared blocks of either 300 or 600 μm in lateral size. Blocks were placed following a grid of 300 or 600 μm spacing, so that there was no overlap between blocks and the spatial dimensions of the resulting designs were all multiple of the basic square dimensions. The blocks were laid on a 20 × 20 mm^2^ area and occupied 15% of it. PDMS (Sylgard 184, Ellsworth Adhesives) with a mixture of 90% base and 10% curing agent was poured on the printed circuit board and cured at 90°C for 2 h (Figure S1C). The PDMS was then gently removed, shaping a topographical relief in which the copper and fiberglass on the board corresponded to depressions and crevices on the PDMS, respectively.

PDMS discs 6 mm in diameter and typically 1 mm thick were then pierced used using stainless steel punchers (Bahco 400.003.020), carefully washed, dried, and attached to glass coverslips (#1 Marienfeld-Superior). A coverslip contained two PDMS discs (Figure S1D). Different sets of coverslips containing PDMS were prepared and sterilized in an autoclave (Selecta 4002515), which in turn strongly bond the PDMS to the glass surface. A detail of the ‘Tracks’ and ‘Squares’ designs ready for culturing are provided in Figure S1E. The bright-field images are accompanied of simple sketches to clarify which are the top and down areas of the designs. Since the copper in the printed board is very smooth, the corresponding PDMS depressions were transparent when observed under bright-field microscopy, whereas the slight roughness of the fiberglass led to PDMS crevices that appeared opaquer. These slight differences in opacity did not affect calcium fluorescence imaging on neurons over the relief. To compare neuronal network dynamics with and without topography, flat PDMS discs were also prepared on a plastic petri dishes and pierced with the aforementioned punchers. These neuronal cultures are referred to as ‘controls’ in the main text.

#### Immunocytochemistry

Neuronal cultures were fixed with 4% PFA (Sigma) for 15 min at room temperature, rinsed with PBS and incubated with blocking solution containing 0.03% Triton (Sigma) and 5% Normal Donkey Serum (Jackson Immunoresearch) in PBS for 45 min at room temperature. Primary antibodies against neuronal cytoskeleton (β3-Tubulin and MAP2) were applied diluted in blocking solution and incubated overnight at 4°C. Alexa488-conjugated secondary antibody against mouse was diluted in blocking solution and incubated for 90 min at room temperature. For astrocytic staining, samples were post-fixed again and incubated with anti-GFAP antibody directly conjugated with Cy3 overnight at 4°C. Then, cultures were rinsed with PBS and mounted using DAPI-fluoromount–G (ShouternBiotech). Immunocytochemical images were acquired on a Zeiss confocal microscope (LSM-880).

#### Calcium imaging

Spontaneous activity in neuronal cultures grown on PDMS topographical substrates was recorded daily at 25°C along two weeks, from DIV 7 (the onset of strong GCAMP6s calcium signal) to 21 (the beginning of culture degradation). Since monitoring the same culture was crucial, the set of prepared wells was inspected in detail before any recording, and cultures that did not have a homogeneous distribution of neurons or that were inactive at DIV 7 were discarded.

For the selected cultures, the 4-well plate in which they sit was mounted on a Zeiss Axiovert C25 inverted microscope equipped with a high-speed CMOS camera (Hamamatsu Orca Flash 4.1). The combination of a 2.5× objective and an optical zoom allowed for the visualization of an entire 6 mm culture with a spatial resolution of 5.9 μm/pixel, an image size of 1,024×1,024 pixels, and 8-bit gray scale format. Spontaneous activity recordings were carried out for 30 min at 100 images/s and repeated every 24 h. The orientation of a given culture relative to the camera was maintained along the 2-week culture evolution to facilitate data analysis.

#### Calcium imaging data analysis and activity events detection

Fluorescence recordings were analyzed with the custom-made software Netcal run in MATLAB.^34^^,^^68^ For convenience, and since the scope of the study was to investigate collective behavior, a set of 1,300 Regions of Interest (ROIs) were laid out on the image (Figure S2A). The ROIs shaped a grid centered at the culture and extending its entire circular shape of 6 mm diameter. A ROI had a typical size of 150 × 150 μm and contained about 5–10 neurons. The average fluorescence intensity of each ROI i along the 30 min recording duration was then extracted, and the obtained fluorescence trace $F_{i}\left(t\right)$ was corrected from drifts and normalized as $\Delta FF_{i}\;(t)\equiv \left(F_{{}_{i}}(t)-F_{i,0}\right)/F_{i,0}$, where $F_{i,0}$ is the basal fluorescence level (without neuronal activity) (Figure S2B).

Fluorescence data of each ROI was converted into time series of neuronal activity by using the Schmitt trigger method, which accepts a sharp change in fluorescence as an activity episode whenever the fluorescence stays elevated for at least 100 ms between a lower and a higher threshold.^70^ The two thresholds were necessary to prevent that camera noise or other artifacts were identified as activity events. The Schmitt trigger method captured the onset time of activity in each ROI, independently on the amplitude of the fluorescence peak as far as it was sufficiently high. The train of detected events, extended to all ROIs in the network, was visualized as raster plots (Figure S2C) and framed the core dataset for in-depth analysis of the neuronal cultures.

#### Population activity, network bursts, and distribution of burst amplitudes

The population activity A quantified the capacity of the neurons in the network to exhibit coordinated activity, i.e., the coordinated activation of a fraction of the network within a window of 100 ms. The population activity was computed as the fraction of ROIs in the network that activated together without repetition in a sliding window 1 s wide and 0.1 s step. A varied between 0 (no activity) and 1 (full network activation). Sharp peaks in A identified strong coordinated activity and were denoted as *network bursts*. These bursts were deemed significant when their amplitude $A_{b}$ verified $A_{b}>\mu_{bgnd}+3\cdot SD_{bgnd}$, where $\mu_{bgnd}$ and $SD_{bgnd}$ are the mean and SD of background activity. In general, for most of the experiments, significant bursts were those with $A_{b}>0.1$, *i*.*e*., 10% of the network active. All significant burst amplitudes $A_{b}$, across realizations and for a given experimental condition, were pooled together to build the distribution of amplitudes. These distributions were finally compared between the different topographical designs and the control, flat PDMS condition.

#### Dynamical richness

The dynamical richness Θ provides a measure for the spatiotemporal variability of network activity, *i*.*e*., the existence of a broad range of coactivation patterns and dynamical states, and is defined as^22^:

$$\Theta =\left(1-\frac{n_{b}}{2\left(n_{b}-1\right)}\sum_{\mu =1}^{n_{b}}\left|p_{\mu }\left({CC}_{ij}\right)-\frac{1}{n_{b}}\;\right|\right)\left(1-\frac{n_{b}}{2\left(n_{b}-1\right)}\sum_{\mu =1}^{n_{b}}\left|p_{\mu }\left(A_{b}\right)-\frac{1}{n_{b}}\;\right|\right)\text{,}$$

where $p\left({CC}_{ij}\right)$ is the distribution of Pearson’s correlation coefficients between the activity trains of all pairs of ROIs i and j, $p\left(A_{b}\right)$ the distribution of burst sizes $A_{b}$, |·| denotes the absolute value and $n_{b}=20$ is the number of bins used for estimating the distributions. Θ varies between 0 (no richness) and 1 (full richness). Conceptually, Θ≃0 corresponds to a scenario of random activity or coherent, whole-network activations, whereas Θ≃1 corresponds to a network state in which neurons coactivate in groups of richly varying size and temporal occurrence.

#### Bursts’ analysis as spatiotemporal fronts

Network bursts propagated throughout the PDMS surface as spatiotemporal fronts whose structure and velocity depended on the underpinned topographical design. The propagation of a given burst was depicted in Figure 2A as an image plot, in which each active ROI was shown in the Euclidean x−y space and colored according to its activation time. Dark colors represented those regions that activated first, and yellow-white colors those that activated the latest. Inactive ROIs or regions of the map without ROIs were shown in dark gray. The origin of activity, termed ‘burst initiation point’, was computed by considering a group of 10 ROIs with the shortest activation times and by analyzing all combinations of 4 ROIs within the group, computing for each combination the average inter-ROI Euclidean distance $d_{0}$ and average activation time $t_{0}$. The combination that procured the lowest *d*_*0*_ and shortest $t_{0}$ (termed $t_{0}^{\min}$) was selected as initiator and the ROIs’ centroid $\left(x_{0},y_{0}\right)$ was evaluated. This centroid was ascribed as the burst initiation point and was shown in the image plots of Figure 2A as a gray circle. Finally, the activation times $t_{i}$ of all ROIs i were then shifted according to the origin of activity as $t_{i}^{\prime }=t_{i}-t_{0}$. ROIs with negative time $t_{i}^{\prime }$ values were set to 0.

The collection of burst initiation points for each experiment was further analyzed to study their spatial distribution and quantify the tendency for the spontaneous activity to start in the same area of the culture. Following,^66^^,^^71^ the distribution of points was converted to a probability density function of burst initiation, from which the Gini coefficient λ was extracted as a measure of activity focalization. λ→1 indicated a tendency toward a strongly focalized initiation in the same spot, whereas λ→0 indicated a tendency toward a homogeneous distribution of initiation across the culture.

#### Similarity of spatiotemporal fronts

The richness of activity repertoire in a culture was quantified by analyzing the similarity among bursts’ spatiotemporal structure. A coarse approach (Figure 2B) consisted in plotting the bursts as blue-red color bands, where the width of the band is the number of active ROIs and its color patterning indicates the propagation of activity. Each band has its own ROI ordering, from the one that started activity to the last. Bursts with similar band widths and color schemes indicate a comparable propagation structure, *i*.*e*., similar number of active ROIs and temporal evolution. A more refined approach was carried out as follows. First, each burst i was treated as a vector $v_{i}$ whose elements contained the activation time of each ROI. Non-active ROIs were set to −1. All bursts’ vectors preserved the same indexing of ROIs, *i*.*e*., a given position in all vectors contained the activation time of the same ROI. Second, for each pair of bursts’ vectors $v_{i}$ and $v_{j}$, the number of common active ROIs (non-negative entries in both vectors) was determined, and this number was divided by the burst that had the largest number of active ROIs. This procured a ‘common ROIs’ matrix $C=c_{ij}$, in which bursts that shared most of the ROI indexes had $c_{ij}\to 1$, otherwise $c_{ij}\to 0$. Third, cross correlation was carried out between all pairs of bursts’ vectors but using only those ROIs that were active in both vectors, leading to a correlation coefficients matrix $R=r_{ij}$. Values of $r_{ij}\to 1$ indicated pairs of bursts with almost identical spatiotemporal structure (same active ROIs and propagation times), while r_*ij*_ → 0 indicated bursts that shared few ROIs or that propagated in a completely different way. A final matrix **S** of similarity among burst pairs was obtained as the element-wise multiplication of ***C*** and ***R***, *i*.*e*., S=C∘R. To classify the bursts and visualize the matrix ***S***, community structure analysis was carried out in ***S*** using a fast implementation of the Louvain algorithm,^72^ procuring a new matrix whose elements were ordered according to the detected communities (Figure 2C). For clarity of data visualization, bursts’ indexes in Figure 2B were also sorted according to the detected communities in Figure 2C.

#### Velocity of propagating fronts

The propagation speed of activity fronts was analyzed by computing the Euclidean distance $\rho_{i}$ of each ROI i to the origin of activity $\left(x_{0},y_{0}\right)$ and by plotting next $\rho_{i}$ as a function of $t_{i}^{\prime }$, the activation times relative to the origin of activity. An estimation of the global propagation velocity was obtained by the slope of a linear fit $\rho_{i}\left(t_{i}^{\prime }\right)$, and with the intercept forced at (0,0). Only fits with Pearson’s regression coefficients r≥0.95 were accepted, with the best fits corresponding to bursts that propagated as neat circular fronts^66^ and that were typically observed in control cultures. The determination of parallel and transverse velocities for the ‘tracks’ configuration was carried out similarly but after selecting either a row of ROIs in the culture (parallel velocity) or a column (transverse velocity).

#### Effective connectivity

Causal relationships among pairs of ROIs’ activity trains were computed by using a modified version of Generalized Transfer Entropy (GTE)^29^^,^^30^^,^^73^ run in MATLAB. Binarized vectors for the 30 min activity trains (‘1’ for the presence of a spike, ‘0’ for absence) were constructed using a time bin of 20 ms, and an effective connection from ROI I to ROI J ($TE_{I\to J}$) was established whenever the information contained in I significantly increased the capacity to predict future states of J. Instant feedback was present, and Markov Order was set to 2^73^. The significance threshold z for effective connections was established by comparing the transfer entropy estimate $TE_{I\to J}$ with the joint distribution of all input X to J and output I to Y (for any X and Y), as

$$z=\frac{TE_{I\to J}-\left\langle TE_{joint}\right\rangle }{\sigma_{joint}}\text{,}$$

where $\left\langle TE_{joint}\right\rangle$ is the average value of the joint distribution and $\sigma_{joint}$ its SD. Significant connections were then set as those with z≥2. This threshold was considered optimal to capture effective communication both at global and local scales,^30^*i*.*e*., whole-network collective activity and interactions at the PDMS relief level. Significant TE scores were finally set to 0 (absence of connection) or 1 (connection present), shaping directed yet unweighted connectivity matrices. These matrices were visualized in the form of network maps with Gephi.^69^

The effective connectivity data was used to computer a number of network measures as well as basic descriptors related to the intrinsic spatial embedding of the cultures. The latter included the *Euclidean distance between effectively connected ROIs*, $d_{ij}$ (in mm), which was simply given by

$$d_{ij}={\left[{\left(x_{j}–x_{i}\right)}^{2}+{\left(y_{j}–y_{i}\right)}^{2}\right]}^{1/2}\text{,}$$

where $x_{i}$, $y_{i}$ are the spatial coordinates of a ROI i on the culture’s surface; and the *angle between effectively connected neurons* $\theta_{ij}$, which was computed as the angle (in degrees) formed by the vectors of the effective connection i→j and the positive X axis of the culture’s surface. An angle of $90^{\circ }$ or ${-90}^{\circ }$ in the tracks configuration, for instance, indicates that neuronal communication occurs preferentially along the direction of the tracks. For both descriptors, the probability distribution functions were computed by building the histograms of either d (bin size 0.2 mm) or θ (bin size $10^{\circ }$) and normalizing them.

We arbitrarily set an effective connection with Euclidean distance d to be considered ‘long range’ when d was larger than 75% the diameter of the culture. Thus, for our 6 mm diameter cultures, this ‘long range’ threshold corresponded to 4.5 mm.

It is important to note that effective connectivity informs about the flow of neuronal communication across the culture. It may be related, but do not directly reflect, the underpinned structural connectivity, e.g., axonal lengths or synaptic strengths.

#### Network measures

Effective connectivity matrices were analyzed using the ‘Brain Connectivity Toolbox’,^74^ run in MATLAB, to quantify their topological organization. The following network measures were used.

##### Degree distribution p(k)

For directed networks, one must consider both the ingoing (in-degree) $k_{i}^{in}$ and outgoing (out-degree) $k_{i}^{out}$ of neuron i. Here, however, we considered only the in-degree distribution since in a previous numerical study^30^ we showed that $k_{in}$ better captures the differences among patterned cultures. The probability distribution function $p\left(k^{in}\right)$ of in-degree values $k^{in}$ for a given network was obtained by computing the histogram of observed $k^{in}$ values with bin size 2 and normalizing it.

##### Clustering coefficient CC

*CC* measures the cohesiveness of the network at a local scale, with large *CC* values reflecting groups of neurons that strongly interact to one another. *CC* is computed as the ratio between the number of triangles with i as one vertex and the number of all possible triangles that i could form, as described in.^29^^,^^30^^,^^73^ The probability distribution function of CC values was evaluated by computing the histogram of observed *CC* values in a given network with bin size 0.04 and normalizing it.

##### Global efficiency $G_{E}$ and Local efficiency $L_{E}$

$G_{E}$^75^ accounts for the capacity of neurons to exchange information across the entire network, and is defined as

$$G_{E}=\frac{1}{N\left(N-1\right)}\sum_{0\leq i,j\leq N}\frac{1}{d\left(i,j\right)}\text{,}$$

where N is the number of ROIs and d(i,j) is the length of the shortest topological path connecting ROIs i and j, with non-connected ROIs procuring d(i,j)=∞. $G_{E}≃0$ indicates that any ROI poorly communicates with any other in the network, while $G_{E}≃1$ indicates that there is a strong capacity for information exchange at the whole-network scale.

The local efficiency $L_{E}$ of each node of the network i was computed in an equivalent manner but considering only the subgraph formed by node i and its connected neighborhood. High $L_{E}$ values indicate the tendency for strong local communication in the network. The probability distribution function of $L_{E}$ values was evaluated but computing the histogram of observed $L_{E}$ values in a given network with bin size 0.04 and normalizing it.

##### Modularity index Q

The modularity index Q^72^ accounts for the tendency of neurons to form functional modules, *i*.*e*., groups of neurons that are more connected within their groups than with neurons in other groups, and is defined as

$$Q=\frac{1}{2m}\sum_{0\leq i,j\leq N}\left(A_{ij}-\frac{k_{i}k_{j}}{2m}\right)\delta \left(c_{i},c_{j}\right)\text{,}$$

where N is the number of ROIs, $A_{ij}$ represents the weight of the connection between i and j, $k_{i}=\sum_{j=1}^{N}A_{ij}$ is the sum of the weights of the connections attached to neuron i, $c_{i}$ is the community to which neuron i belongs, $m=\left(1/2\right)\sum_{i,j=1}^{N}A_{ij}$, and δ(u,v) is the Kronecker Delta with δ(u,v)=1 for u=v and 0 otherwise. Optimal community structure was computed using the Louvain algorithm.^72^ Q ranges between 0 (the entire network shapes a unique module) and 1 (each ROI is an isolated module). Values of Q≳0.3 indicate the existence of modules in the network with a varying number of ROIs and interconnected to one another.

##### Betweenness centrality BC

The *betweenness centrality* of a node i describes its importance in routing information flow across a network. The higher the BC, the larger the number of shortest path in a network that pass through it. BC is determined as the fraction of shortest paths between any pair of nodes j,k in the network that pass through the node i, and is given by

$$BC_{i}=\sum_{j\;\neq \;k}^{N}\frac{n_{jk}\left(i\right)}{n_{jk}}\text{,}$$

where $n_{jk}$ is the number of shortest paths that link j to k, and $n_{jk}\left(i\right)$ is the number of shortest paths connecting j with k that travel through i.

In all studied data, the set of BC values for a given network were scaled by the maximum, so that data was normalized between 0 and 1. The probability distribution function of BC values was then evaluated but computing the histogram of observed BC values in a given network with bin size 0.04 and normalizing it.

#### Spatial compactness of functional modules

Compactness C refers to the property of objects to exhibit a minimum perimeter P for a given area S, and is mathematically measured through the Polsby-Popper test, $C=4\pi S/P^{2}$, with C=0 for a lack of compactness, *e*.*g*., randomly scattered spots, and 1 for a circle, the most compact shape. The compactness of the effective networks shown in Figure 5 was determined as follows. For each functional module, its participating ROIs were drawn as solid white squares on a black background. ROIs were laid down following a grid, so that two adjacent ROIs shaped a solid rectangle. The final white object containing all participating ROIs was then processed to eliminate single black squares surrounded by white regions. This was necessary to prevent that few empty regions could dominate the perimeter of the object. The compactness $C_{i}$ for the object (functional module) i was then computed. To correct for the artifact associated with the square shape of the ROIs, which increased the perimeter of the object and procured lower compactness than expected, a reference compactness for the entire culture $C_{culture}$ was also determined by using all original ROIs of the experiment and that, by construction, shaped a circle. Typically, $C_{culture}≃0.65$, smaller than the expected value of 1 associated with a perfect circle. Thus, for each functional module, its compactness was corrected as $C_{i}^{*}=C_{i}/C_{culture}$. The compactness values shown in Figure 5 were finally obtained, for a given culture, as $C^{*}=\left(1/n_{f}\right)\sum_{i}C_{i}^{*}$, with $n_{f}$ the number of functional modules.

#### Numerical simulations

Numerical simulations of $N\approx 10^{4}$ spatially embedded neurons were carried out following the biologically realistic construction of^29^^,^^57^^,^^66^ and.^29^^,^^30^^,^^73^ Neurons were modeled as Izhikevich, single spiking integrate-and-fire units with added pre-synaptic depression dynamics. The latter accounted for the depletion of neurotransmitter following repetitive firing. Excitatory neurons comprised 80% of the network and the remaining 20% were inhibitory. The excitatory or inhibitory role of the neurons in the network was randomly chosen. The neurons at rest received no inputs, except for a white noise intrinsic to each neuron. Each simulation was carried out for $1.2\times 10^{5}$ time-steps, corresponding to a simulated time of 120 s. The spatially embedded networks were constructed by placing neurons randomly in a circular area with a diameter of 6 mm, as in the experiments, resulting in a uniform density of 400 neurons/mm^2^. Next, for each neuron, axonal growth was modeled by successively placing 10 μm line segments, each with an angle drawn form a zero mean Gaussian distribution with 0.1 radians SD with respect to the direction of the previous segment, up to a total length drawn for each neuron independently from a Rayleigh distribution with mean 1 mm.^29^^,^^57^^,^^66^ This resulted in nearly straight axons. A circular area around each neuron with a radius drawn from a Gaussian distribution with mean 150 and 40 μm SD functioned as the dendritic tree. Once the axon of a neuron crossed into this dendritic area of another neuron, a connection from the first to the latter was established with a probability 0.1. The topographical PDMS patterns were implemented by placing virtual borders in the culture area corresponding to either bottom-to-top or top-to-bottom transitions. When, during the formation of the axons, a line segment was placed such that it crossed one of these borders it would cross with a probability P, and be deflected with the inverse probability 1−P. The crossing probability was dependent on the direction of crossing: for bottom-to-top transitions P=0.6, while for top-to-bottom P=0.8.

Simulations procured raster plots and collective dynamics that qualitatively resembled those from experiments. For connectivity analysis, 1000 neurons were randomly sampled from the raster plots. The *Z* score used was the same as for the experimental data analysis.

#### Ethical approvals

Dissection of rat embryonic cortices and preparation of primary neuronal cultures were carried out in accordance with the regulations of the Ethical Committee for Animal Experimentation of the University of Barcelona (approved ethical order B-RP-094/15–7125 of July 10^th^, 2015) and the laws for animal experimentation of the Generalitat deCatalunya (Catalonia, Spain).

### Quantification and statistical analysis

Statistical and graphical analyses were conducted with Origin 9.1 and MATLAB 2018a software packages. One–way ANOVA was used to analyze the non-normally distributed data of Figure 1D, and results verified with the Mann-Whitney test. Student’s *t-*test was used to analyze the normally distributed data of Figures 4 and 5. Statistical significance was designated at p<0.05 for all analyses. When appropriate, data were represented and examined via boxplots.

## Data Availability

• Experimental and numerical data have been deposited at Mendeley Data and are publicly available as of the date of publication. The DOI is listed in the key resources table. • This paper does not report original code. • Any additional information required to reanalyze the data reported in this paper is available from the lead contact upon request.

## References

[1] Blankenship A.G., Feller M.B. (2010). Mechanisms underlying spontaneous patterned activity in developing neural circuits. Nat. Rev. Neurosci..

[2] Suárez L.E., Markello R.D., Betzel R.F., Misic B. (2020). Linking structure and function in macroscale brain networks. Trends Cogn. Sci..

[3] Park H.-J., Friston K. (2013). Structural and functional brain networks: from connections to cognition. Science.

[4] Finc K., Bonna K., He X., Lydon-Staley D.M., Kühn S., Duch W., Bassett D.S. (2020). Dynamic reconfiguration of functional brain networks during working memory training. Nat. Commun..

[5] Meunier D., Lambiotte R., Bullmore E.T. (2010). Modular and hierarchically modular organization of brain networks. Front. Neurosci..

[6] Sporns O. (2013). Network attributes for segregation and integration in the human brain. Curr. Opin. Neurobiol..

[7] Deco G., Tononi G., Boly M., Kringelbach M.L. (2015). Rethinking segregation and integration: contributions of whole-brain modelling. Nat. Rev. Neurosci..

[8] Bullmore E., Sporns O. (2012). The economy of brain network organization. Nat. Rev. Neurosci..

[9] Aebersold M.J., Dermutz H., Forró C., Weydert S., Thompson-Steckel G., Vörös J., Demkó L. (2016). “Brains on a chip”: towards engineered neural networks. TrAC7 Trends Anal. Chem..

[10] Wheeler B.C., Brewer G.J. (2010). Designing Neural Networks in Culture: experiments are described for controlled growth, of nerve cells taken from rats, in predesigned geometrical patterns on laboratory culture dishes. Proc. IEEE. Inst. Electr. Electron. Eng..

[11] Millet L.J., Gillette M.U. (2012). New perspectives on neuronal development via microfluidic environments. Trends Neurosci..

[12] Bonifazi P., Difato F., Massobrio P., Breschi G.L., Pasquale V., Levi T., Goldin M., Bornat Y., Tedesco M., Bisio M. (2013). In vitro large-scale experimental and theoretical studies for the realization of bi-directional brain-prostheses. Front. Neural Circuits.

[13] Poli D., Pastore V.P., Massobrio P. (2015). Functional connectivity in in vitro neuronal assemblies. Front. Neural Circuits.

[14] Marconi E., Nieus T., Maccione A., Valente P., Simi A., Messa M., Dante S., Baldelli P., Berdondini L., Benfenati F. (2012). Emergent functional properties of neuronal networks with controlled topology. PLoS One.

[15] Brofiga M., Pisano M., Raiteri R., Massobrio P. (2021). On the road to the brain-on-a-chip: a review on strategies, methods, and applications. J. Neural. Eng..

[16] Grienberger C., Konnerth A. (2012). Imaging calcium in neurons. Neuron.

[17] Kim R., Joo S., Jung H., Hong N., Nam Y. (2014). Recent trends in microelectrode array technology for in vitro neural interface platform. Biomed. Eng. Lett..

[18] Neto E., Leitão L., Sousa D.M., Alves C.J., Alencastre I.S., Aguiar P., Lamghari M. (2016). Compartmentalized microfluidic platforms: the unrivaled breakthrough of in vitro tools for neurobiological research. J. Neurosci..

[19] Forró C., Thompson-Steckel G., Weaver S., Weydert S., Ihle S., Dermutz H., Aebersold M.J., Pilz R., Demkó L., Vörös J. (2018). Modular microstructure design to build neuronal networks of defined functional connectivity. Biosens. Bioelectron..

[20] Park M.U., Bae Y., Lee K.S., Song J.H., Lee S.M., Yoo K.H. (2021). Collective dynamics of neuronal activities in various modular networks. Lab Chip.

[21] Bisio M., Bosca A., Pasquale V., Berdondini L., Chiappalone M. (2014). Emergence of bursting activity in connected neuronal sub-populations. PLoS One.

[22] Yamamoto H., Moriya S., Ide K., Hayakawa T., Akima H., Sato S., Kubota S., Tanii T., Niwano M., Teller S. (2018). Impact of modular organization on dynamical richness in cortical networks. Sci. Adv..

[23] Pașca S.P. (2018). The rise of three-dimensional human brain cultures. Nature.

[24] Zhuang P., Sun A.X., An J., Chua C.K., Chew S.Y. (2018). 3D neural tissue models: from spheroids to bioprinting. Biomaterials.

[25] Choi J.S., Lee H.J., Rajaraman S., Kim D.H. (2021). Recent advances in three-dimensional microelectrode array technologies for in vitro and in vivo cardiac and neuronal interfaces. Biosens. Bioelectron..

[26] Downes J.H., Hammond M.W., Xydas D., Spencer M.C., Becerra V.M., Warwick K., Whalley B.J., Nasuto S.J. (2012). Emergence of a small-world functional network in cultured neurons. PLoS Comput. Biol..

[27] Schroeter M.S., Charlesworth P., Kitzbichler M.G., Paulsen O., Bullmore E.T. (2015). Emergence of rich-club topology and coordinated dynamics in development of hippocampal functional networks in vitro. J. Neurosci..

[28] Okujeni S., Egert U. (2019). Self-organization of modular network architecture by activity-dependent neuronal migration and outgrowth. Elife.

[29] Tibau E., Ludl A.A., Rudiger S., Orlandi J.G., Soriano J. (2020). Neuronal spatial arrangement shapes effective connectivity traits of in vitro cortical networks. IEEE Trans. Netw. Sci. Eng..

[30] Ludl A.A., Soriano J. (2020). Impact of physical obstacles on the structural and effective connectivity of in silico neuronal circuits. Front. Comput. Neurosci..

[31] Wagenaar D.A., Pine J., Potter S.M. (2006). An extremely rich repertoire of bursting patterns during the development of cortical cultures. BMC Neurosci..

[32] Estévez-Priego E., Teller S., Granell C., Arenas A., Soriano J. (2020). Functional strengthening through synaptic scaling upon connectivity disruption in neuronal cultures. Netw. Neurosci..

[33] Teller S., Estévez-Priego E., Granell C., Tornero D., Andilla J., Olarte O.E., Loza-Alvarez P., Arenas A., Soriano J. (2020). Spontaneous functional recovery after focal damage in neuronal cultures. eNeuro.

[34] Fernández-García S., Orlandi J.G., García-Díaz Barriga G.A., Rodríguez M.J., Masana M., Soriano J., Alberch J. (2020). Deficits in coordinated neuronal activity and network topology are striatal hallmarks in Huntington’s disease. BMC Biol..

[35] Teller S., Tahirbegi I.B., Mir M., Samitier J., Soriano J. (2015). Magnetite-Amyloid-β deteriorates activity and functional organization in an in vitro model for Alzheimer’s disease. Sci. Rep..

[36] Shein-Idelson M., Ben-Jacob E., Hanein Y. (2011). Engineered neuronal circuits: a new platform for studying the role of modular topology. Front. Neuroeng..

[37] Sukenik N., Vinogradov O., Weinreb E., Segal M., Levina A., Moses E. (2021). Neuronal Circuits Overcome Imbalance in Excitation and Inhibition by Adjusting Connection Numbers. Proc. Natl. Acad. Sci. USA.

[38] Isaacson J.S., Scanziani M. (2011). How inhibition shapes cortical activity. Neuron.

[39] Vensi Basso J.M., Yurchenko I., Simon M., Rizzo D.J., Staii C. (2019). Role of geometrical cues in neuronal growth. Phys. Rev. E.

[40] Li W., Xu Z., Huang J., Lin X., Luo R., Chen C.-H., Shi P. (2014). NeuroArray: a universal interface for patterning and interrogating neural circuitry with single cell resolution. Sci. Rep..

[41] Li S., Kuddannaya S., Chuah Y.J., Bao J., Zhang Y., Wang D. (2017). Combined effects of multi-scale topographical cues on stable cell sheet formation and differentiation of mesenchymal stem cells. Biomater. Sci..

[42] Sumi T., Yamamoto H., Hirano-Iwata A. (2020). Suppression of hypersynchronous network activity in cultured cortical neurons using an ultrasoft silicone scaffold. Soft Matter.

[43] Holloway P.M., Willaime-Morawek S., Siow R., Barber M., Owens R.M., Sharma A.D., Rowan W., Hill E., Zagnoni M. (2021). Advances in microfluidic in vitro systems for neurological disease modeling. J. Neurosci. Res..

[44] Nikolakopoulou P., Rauti R., Voulgaris D., Shlomy I., Maoz B.M., Herland A. (2020). Recent progress in translational engineered in vitro models of the central nervous system. Brain.

[45] Liu W., Fu W., Sun M., Han K., Hu R., Liu D., Wang J. (2021). Straightforward neuron micropatterning and neuronal network construction on cell-repellent polydimethylsiloxane using microfluidics-guided functionalized Pluronic modification. Analyst.

[46] Okujeni S., Kandler S., Egert U. (2017). Mesoscale architecture shapes initiation and richness of spontaneous network activity. J. Neurosci..

[47] Bassett D.S., Sporns O. (2017). Network neuroscience. Nat. Neurosci..

[48] Farahani F.V., Karwowski W., Lighthall N.R. (2019). Application of graph theory for identifying connectivity patterns in human brain networks: a systematic review. Front. Neurosci..

[49] Emiliani V., Cohen A.E., Deisseroth K., Häusser M. (2015). All-optical interrogation of neural circuits. J. Neurosci..

[50] Spanu A., Tedesco M., Martinoia S., Bonfiglio A., Pasquale V., Frega M. (2019). In Vitro Neuronal Networks M. Chiappalone.

[51] Bressloff P.C. (2000). Traveling waves and pulses in a one-dimensional network of excitable integrate-and-fire neurons. J. Math. Biol..

[52] Golomb D., Ermentrout G.B. (1999). Continuous and lurching traveling pulses in neuronal networks with delay and spatially decaying connectivity. Proc. Natl. Acad. Sci. USA.

[53] Feinerman O., Segal M., Moses E. (2005). Signal propagation along unidimensional neuronal networks. J. Neurophysiol..

[54] Jacobi S., Moses E. (2007). Variability and corresponding amplitude-velocity relation of activity propagating in one-dimensional neural cultures. J. Neurophysiol..

[55] Jacobi S., Soriano J., Moses E. (2010). BDNF and NT-3 increase velocity of activity front propagation in unidimensional hippocampal cultures. J. Neurophysiol..

[56] Soriano J., Rodríguez Martínez M., Tlusty T., Moses E. (2008). Development of input connections in neural cultures. Proc. Natl. Acad. Sci. USA.

[57] Tibau E., Valencia M., Soriano J. (2013). Identification of neuronal network properties from the spectral analysis of calcium imaging signals in neuronal cultures. Front. Neural Circuits.

[58] Stiso J., Bassett D.S. (2018). Spatial embedding imposes constraints on neuronal network architectures. Trends Cogn. Sci..

[59] Poli D., Pastore V.P., Martinoia S., Massobrio P. (2016). From functional to structural connectivity using partial correlation in neuronal assemblies. J. Neural. Eng..

[60] Gladkov A., Pigareva Y., Kutyina D., Kolpakov V., Bukatin A., Mukhina I., Kazantsev V., Pimashkin A. (2017). Design of cultured neuron networks in vitro with predefined connectivity using asymmetric microfluidic channels. Sci. Rep..

[61] Brofiga M., Pisano M., Tedesco M., Boccaccio A., Massobrio P. (2022). Functional inhibitory connections modulate the electrophysiological activity patterns of cortical-hippocampal ensembles. Cereb. Cortex.

[62] Kanagasabapathi T.T., Massobrio P., Barone R.A., Tedesco M., Martinoia S., Wadman W.J., Decré M.M.J. (2012). Functional connectivity and dynamics of cortical-thalamic networks co-cultured in a dual compartment device. J. Neural. Eng..

[63] Dauth S., Maoz B.M., Sheehy S.P., Hemphill M.A., Murty T., Macedonia M.K., Greer A.M., Budnik B., Parker K.K. (2017). Neurons derived from different brain regions are inherently different in vitro: a novel multiregional brain-on-a-chip. J. Neurophysiol..

[64] Bang S., Na S., Jang J.M., Kim J., Jeon N.L. (2016). Engineering-aligned 3D neural circuit in microfluidic device. Adv. Healthc. Mater..

[65] Carola G., Malagarriga D., Calatayud C., Pons-Espinal M., Blasco-Agell L., Richaud-Patin Y., Fernandez-Carasa I., Baruffi V., Beltramone S., Molina E. (2021). Parkinson’s disease patient-specific neuronal networks carrying the LRRK2 G2019S mutation unveil early functional alterations that predate neurodegeneration. NPJ Parkinsons Dis..

[66] Orlandi J.G., Soriano J., Alvarez-Lacalle E., Teller S., Casademunt J. (2013). Noise focusing and the emergence of coherent activity in neuronal cultures. Nat. Phys..

[67] Kirwan P., Turner-Bridger B., Peter M., Momoh A., Arambepola D., Robinson H.P.C., Livesey F.J. (2015). Development and function of human cerebral cortex neural networks from pluripotent stem cells in vitro. Development.

[68] Orlandi J.G., Fernández-García S., Comella-Bolla A., Masana M., García-Díaz Barriga G., Yaghoobi M., Canals J.-M., Colicos M.A., Davidsen J., Alberch J. (2017). NETCAL: an interactive platform for large-scale, NETwork and population dynamics analysis of CALcium imaging recordings. Neuroscience.

[69] Bastian M., Heymann S., Jacomy M. (2009). Gephi: an open source software for exploring and manipulating networks. Proc. Int. AAAI Conf. Web Soc. Media.

[70] Grewe B.F., Langer D., Kasper H., Kampa B.M., Helmchen F. (2010). High-speed in vivo calcium imaging reveals neuronal network activity with near-millisecond precision. Nat. Methods.

[71] Faci-Lázaro S., Soriano J., Gómez-Gardeñes J. (2019). Impact of targeted attack on the spontaneous activity in spatial and biologically-inspired neuronal networks. Chaos.

[72] Blondel V.D., Guillaume J.L., Lambiotte R., Lefebvre E. (2008). Fast unfolding of communities in large networks. J. Stat. Mech..

[73] Stetter O., Battaglia D., Soriano J., Geisel T. (2012). Model-free reconstruction of excitatory neuronal connectivity from calcium imaging signals. PLoS Comput. Biol..

[74] Rubinov M., Sporns O. (2010). Complex network measures of brain connectivity: uses and interpretations. Neuroimage.

[75] Latora V., Marchiori M. (2001). Efficient behavior of small-world networks. Phys. Rev. Lett..

