# Supplementary material for: Rich dynamics and functional organization on topographically designed neuronal networks in vitro
Source: iScience. 2022 Nov 26;25(12):105680. doi: 10.1016/j.isci.2022.105680 (PMC9768383; doi:10.1016/j.isci.2022.105680)
Supplement: Document S1. Figures S1–S7 [file mmc1.pdf]

iScience, Volume *25*

Supplemental information

Rich dynamics and functional organization on topographically designed neuronal networks *in vitro*

Marc Montalà-Flaquer, Clara F. López-León, Daniel Tornero, Akke Mats Houben, Tanguy Fardet, Pascal Monceau, Samuel Bottani, and Jordi Soriano

SUPPLEMENTARY FIGURES:

■ **Figure S1.** Preparation of topographical PDMS substrates. Related to Figure 1

■ **Figure S2.** Data analysis. Related to Figure 1.

■ **Figure S3.** Effective connectivity and dynamics. Related to Figure 5.

■ **Figure S4.** Development of effective connections in experiments. Related to Figures 2 and 5.

■ **Figure S5.** Numerical simulations allow to understand the impact of longer axons on effective connectivity. Related to Figures 2 and 5.

■ **Figure S6.** Local network properties for effective connectivity in experimental data at DIV 14. Related to Figure 5.

■ **Figure S7.** The difficulty of extracting structural connectivity from effective one in numerical simulations. Related to Figure 5.

Supplementary Figure S1. Preparation of topographical PDMS substrates. Related to Figure 1

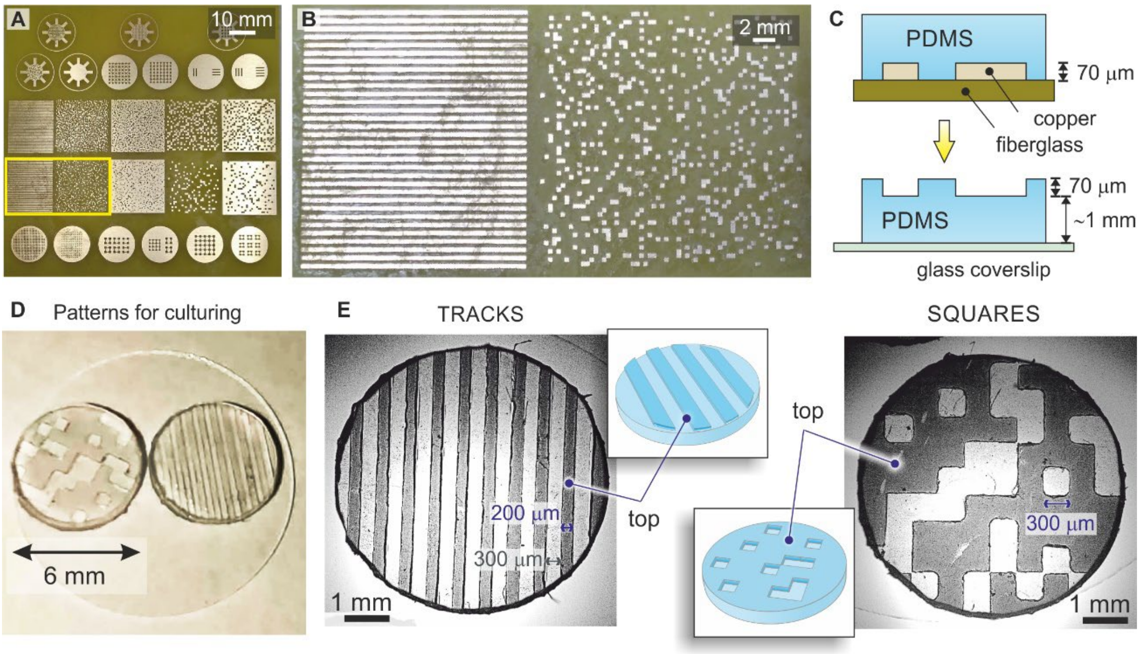

(A) Printed circuit board containing different designs. Copper (bright gold) elevates over the fiberglass (dark green) by 70 μm. The yellow rectangle highlights the designs used in the present study. **(B)** Detail of 'tracks' (left) and 'squares' (right) designs. For tracks, copper tracks elevations are 200 μm thick and are separated by crevices 200 μm wide. For squares, they are 300×300 μm unit size and randomly positioned without overlap and occupying 15% of the available area. **(C)** Sketch of PDMS casting from the printed circuit board. **(D)** A couple of topographical cultures 6 mm in diameter attached to a glass coverslip. **(E)** Bright-field images of the cultures. Transparent and dark areas correspond to PDMS in contact with copper and fiberglass, respectively, and shape the bottom and top regions of the topographical relief.

Supplementary Figure S2. Data analysis. Related to Figure 1

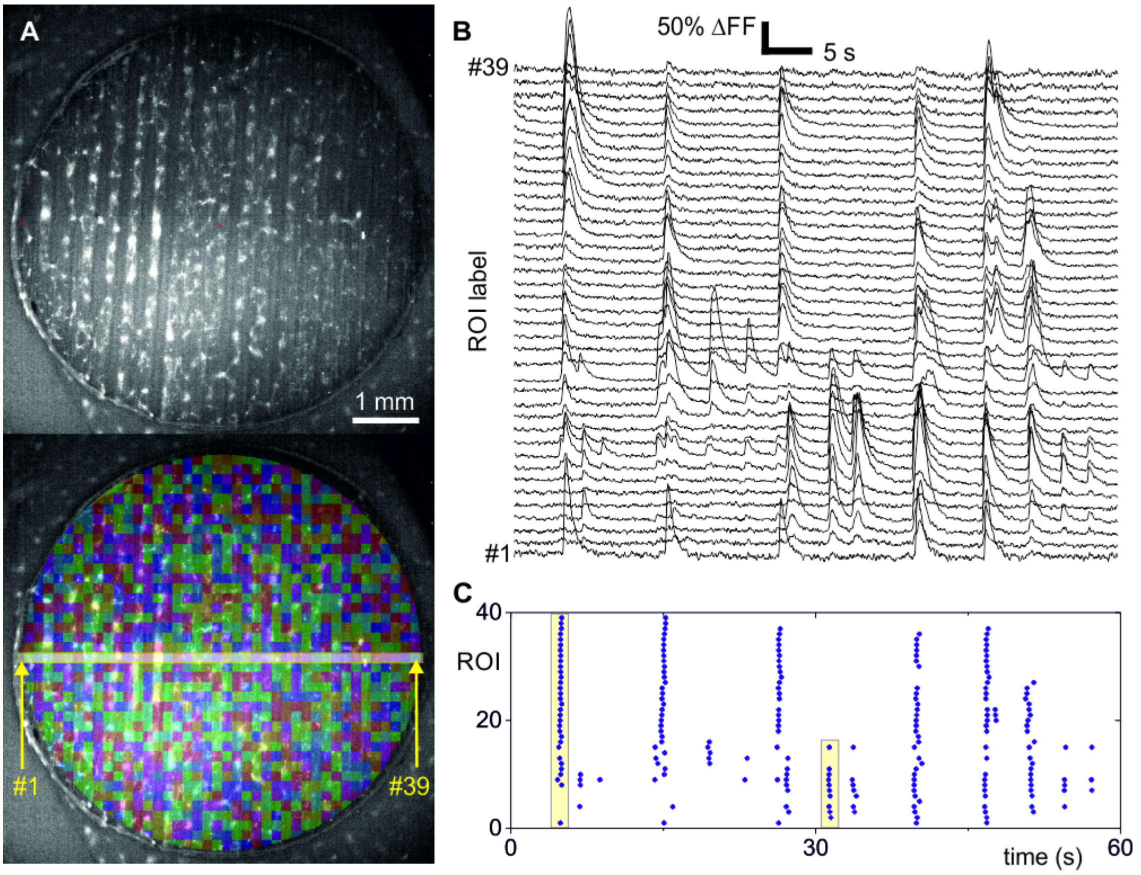

(A) Top, highly contrasted fluorescence image of a 'tracks' culture. Bottom, Regions of Interest (ROIs) set as squared boxes and covering the entire circular culture, with a total number of 1,400 ROIs. The ROIs labeled as 1 to 39 at the center of the culture are used to provide representative fluorescence traces. **(B)** Normalized fluorescence traces of these 39 ROIs along the first minute of the recording, vertically shifted for clarity. Sharp peaks indicate neuronal activity. **(C)** Corresponding raster plot. Examples of coordinated neuronal activity events of large and small sizes are highlighted with yellow boxes.

Supplementary Figure S3. Effective connectivity and dynamics. Related to Figure 5

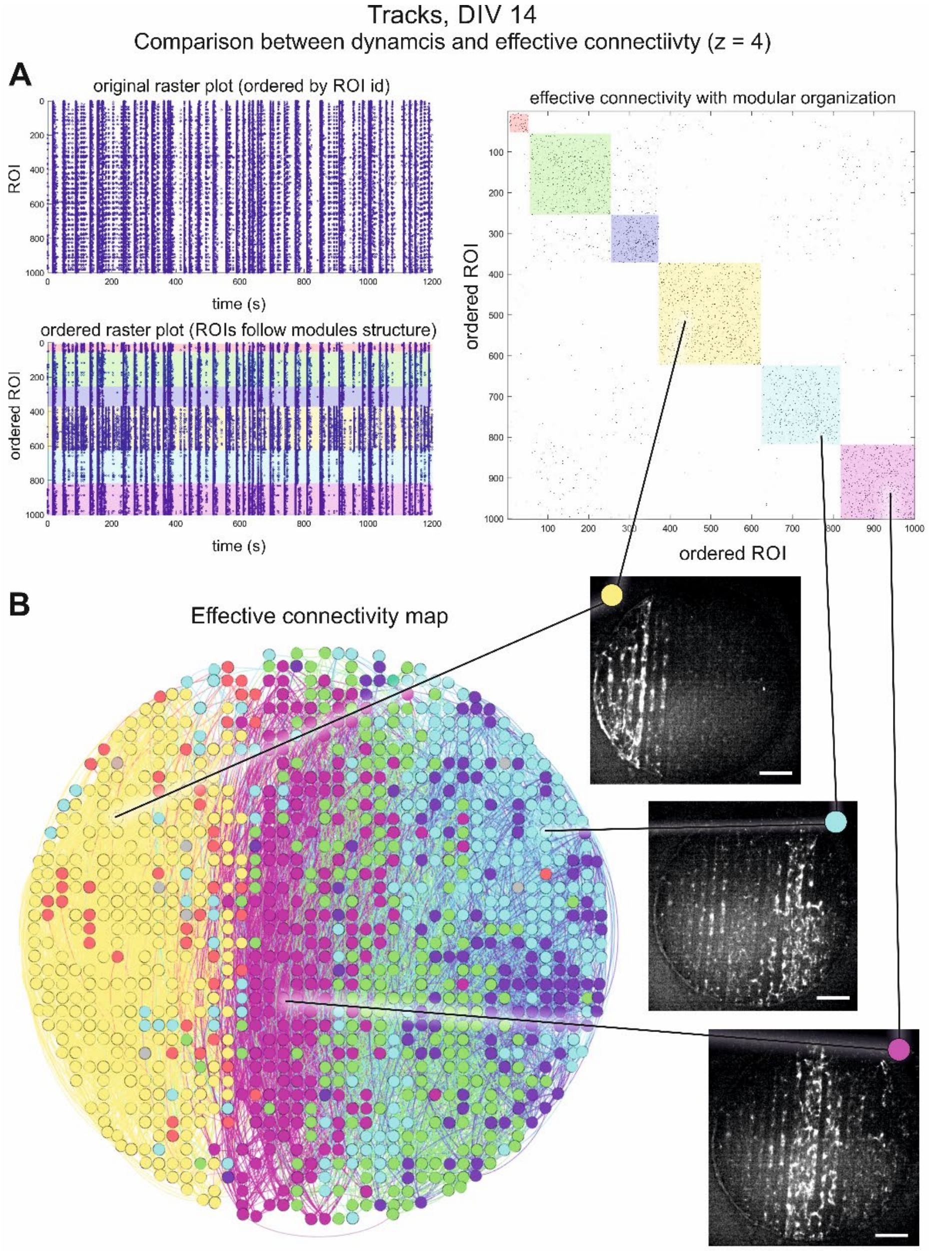

The figure illustrates the relation between effective connectivity and dynamics in the 'tracks' configuration, and for a significance threshold $z = 4$ to highlight activity outside whole network bursts. The experiment is the same as in Figure 5. **(A)** Original raster plot of spontaneous activity (left-top) and effective connectivity matrix depicting the functional modules (right). The reordering of ROI indexes according to the modules shapes a new raster plot (left-bottom) in which dynamic features of the network can be linked to characteristic modules. The yellow module for instance corresponds to a group of ROIs (reordered indexes 400 to 600) with strong spontaneous activity and the presence of collective events that do not exist in the other groups, indicating that functional modules reflect specific activity patterns. **(B)** Network map of effective connections. ROIs are colored according to the functional module they belong to. Some modules, particularly the yellow and the pink, coincide in spatial structure with activity patterns that appear often in the recording (fluorescence snapshots), which illustrates the link between dynamics and functional organization.

Supplementary Figure S4. Development of effective connections in experimental data. Related to Figures 2 and 5

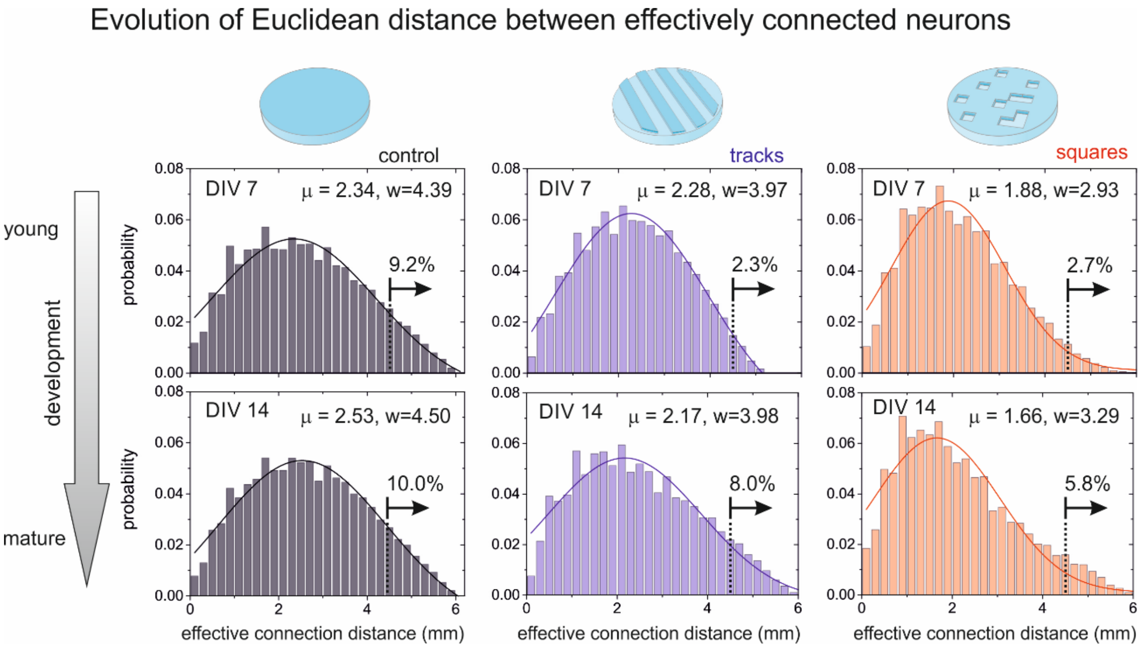

The bar plots show the probability distribution functions of Euclidean distances d between effectively connected ROIs. All distributions show a Gaussian fit that is used to extract the mean μ and width w of the distributions. Long range effective connections are set as those that extend at least 75% of the diameter of the culture, i.e., 4.5 mm (vertical dashed line and arrow). For control, flat PDMS, the fraction of long-range effective connections is about 9% of all existing connections at day *in vitro* (DIV) 7 and remains practically constant along development, a result that is expected since whole-network communication occurs in the form of bursting both in young and mature cultures. For tracks, long-range connections are rare at young (DIV 7) cultures, comprising about 2% of all connections, a result that is consistent with the low occurrence of whole-network bursts. Long-range connections increase 4 times upon maturation and reach levels akin to controls, also in agreement with a stronger overall bursting. A similar trend is observed in squares. Altogether, this analysis shows that a higher presence of bursting events in mature tracks/squares cultures is consistent with stronger whole-network communication and longer range effective connections. Numerical simulations (see Supplementary Figure S5) show that, in turn, an increase in the average length of the axons (simulating development) leads to a stronger whole-network bursting and longer range effective connections.

Supplementary Figure S5. Numerical simulations allow to understand the impact of longer axons on effective connectivity. Related to Figures 2 and 5

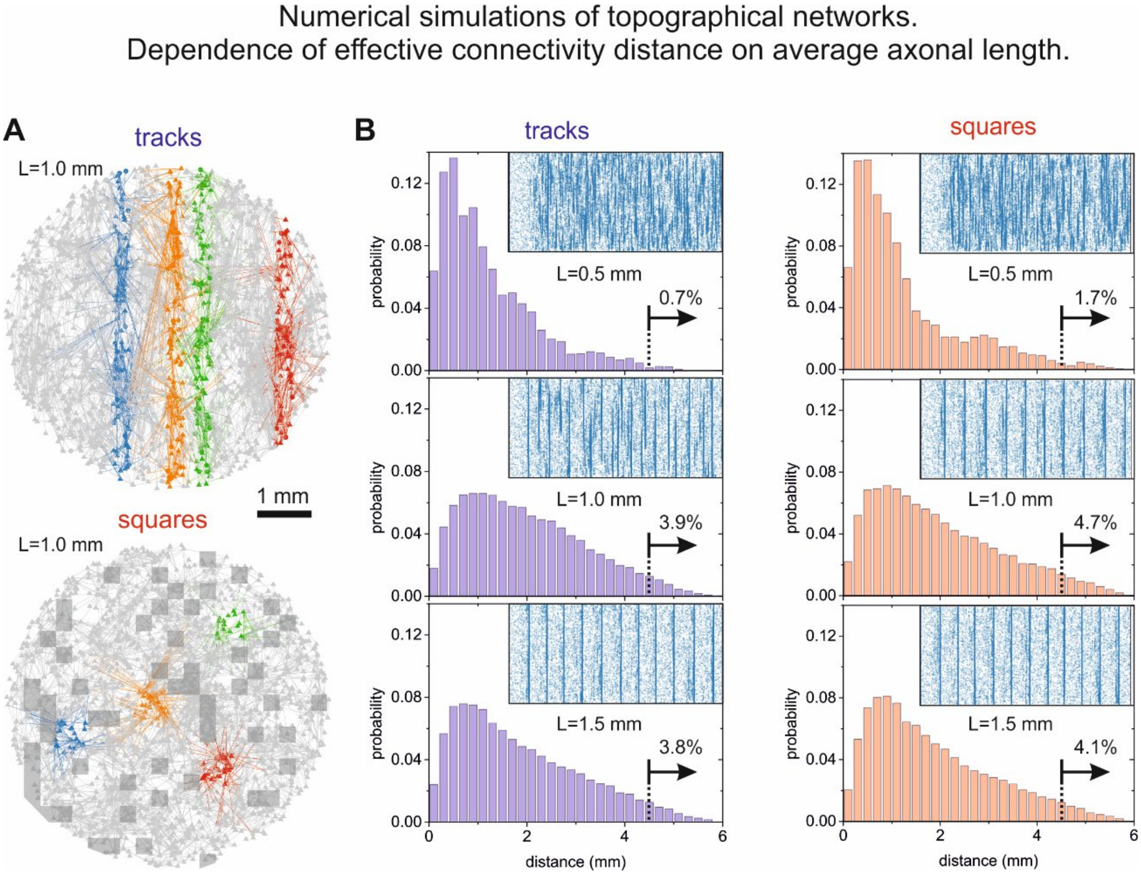

(A) Illustrative network maps of simulated networks with tracks or squares topographical patterns. Neuronal density is 400 neurons/mm^2 and average axonal length is 1 mm. Some neurons and their outgoing connections are color coded according to the structural module they belong to. For the case of the tracks configuration, the modules coincide with the position of the tracks themselves. For the case of squares, the modules form islands in the network. The position of the square valleys is indicated with a gray overlay. **(B)** Probability distribution functions of effective connectivity Euclidean distances, in both tracks and squares configurations, for gradually larger average axonal lengths L that replicate the maturation of the network. The insets show simulated raster plots of spontaneous activity from which effective connectivity data is obtained, using the same GTE inference approach and parameters as in the experiments. For both tracks and squares, short axonal lengths of $L = 0.5$ mm led to rich raster plots qualitatively similar to those observed in the experiments. The corresponding distributions of effective connection distances show that long-range connections (those larger than 4.5 mm, vertical dashed line) are rare, comprising about $1 - 2\%$ of all occurrences, which is consistent with the lack of whole-network activity events in the raster plots. For $L = 1.0$ mm and 1.5 mm, however, long-range connections substantially increase and are about $4 - 5\%$ of all occurrences, a trait that is ascribed to the large-scale communication provided by abundant whole-network bursting. Altogether, the results show that an increase in the Euclidean distance of effective connections is consistent with an increase of longer axonal lengths and, in turn, a higher presence of whole-network bursts.

Supplementary Figure S6. Local network properties for effective connectivity in experimental data at DIV 14. Related to Figure 5

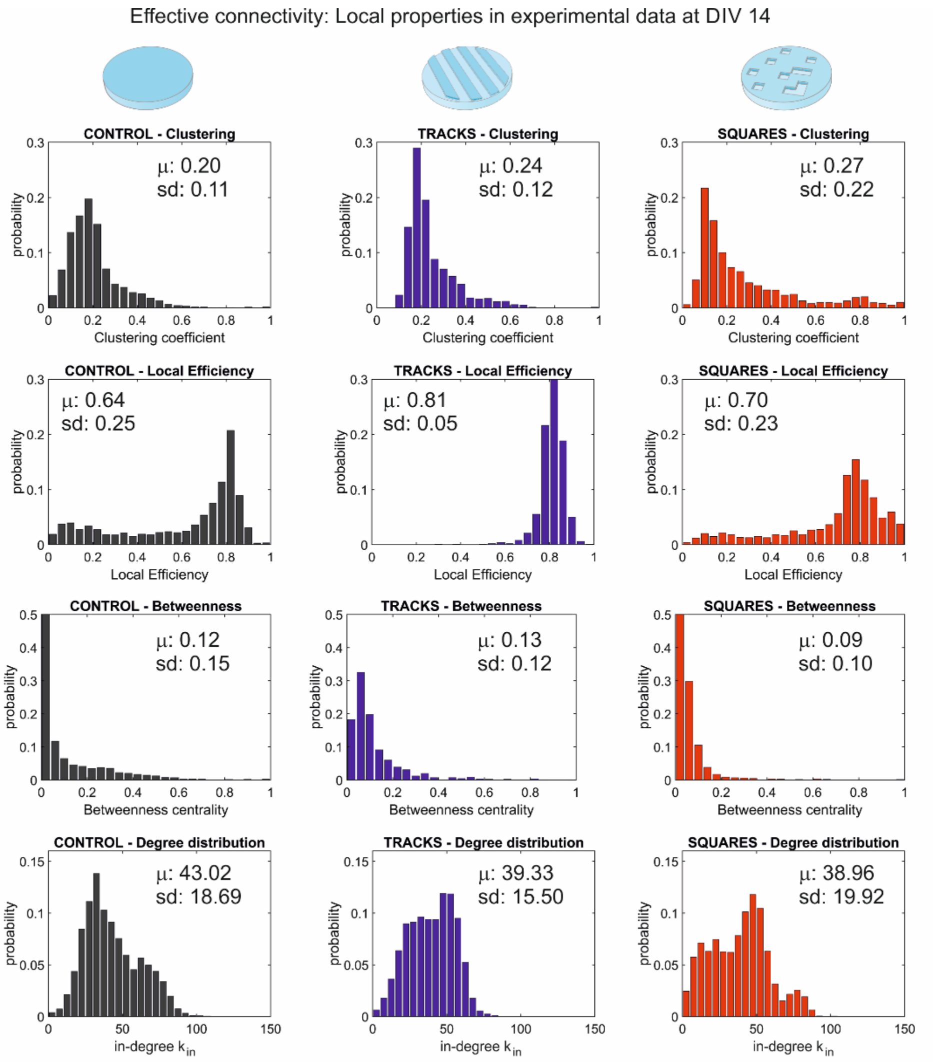

The plots show probability density functions (pdf) of 4 local network metrics derived from the effective connectivity data, comparing control, 'tracks' and 'squares' configurations. All pdfs are averages over the 5-6 experimental realizations per configuration of Figure 5B. The values of μ and sd indicate, respectively, the average of the distributions and their standard deviation. In general, tracks and squares exhibited higher clustering coefficients and local efficiencies than controls, indicating a higher tendency for the topographical patterns to shape local subgraphs and functional communities. Since effective connectivity reflects dynamics, these higher values for tracks/squares may indicate the tendency of neurons to activate in small groups, and not at unison as in controls. The betweenness centrality had similar average values for the 3 configurations, although the peak at zero was less prominent on tracks, possibly indicating that this configuration favored hubs formation, e.g., neurons connecting tracks transversally. The in-degree k_{in} distributions provided similar average value $\langle k_{in} \rangle$ around 40 connections/neuron, but the distributions for tracks and squares were right-skewed (peaked towards high values) as compared to controls, and the distribution for squares was broader than for tracks. It must be noted that the effective connectivity does not fully portray structural connectivity and therefore these comparisons may require further investigations (see also Supplementary Figure S7).

Supplementary Figure S7. The difficulty of extracting structural connectivity from effective one in numerical simulations. Related to Figure 5

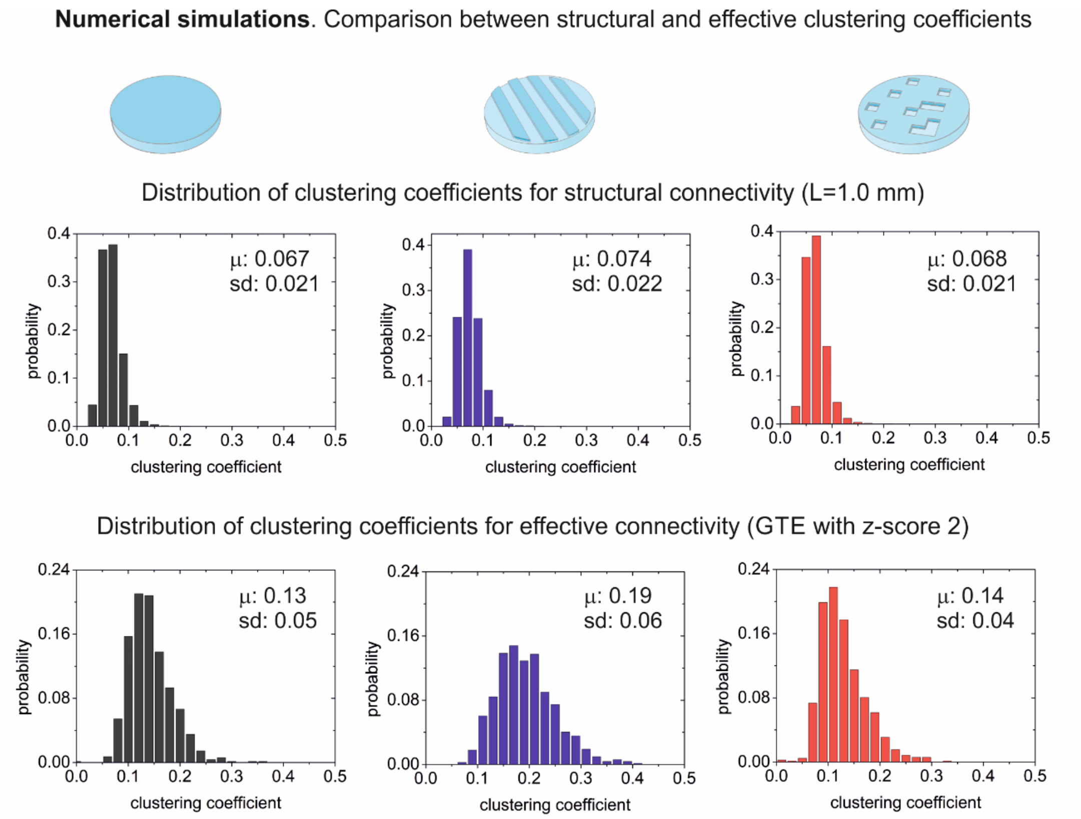

The plots show probability density functions (pdf) of clustering coefficients (CC) values, comparing control, tracks, and squares configurations. Data is drawn from a single numerical repetition. Since data is extracted from simulations, both structural and effective connectivity layouts are accessible. In all three configurations, neuronal density was 400 neurons/mm^2, average axonal length L=1.0 mm, and average in-degree 25. For the structural data, CC values were on average much higher in tracks than in controls or squares, but the two latter were similar, which indicates that it is difficult to strongly imprint local structural features in the squares configuration. For the effective connectivity, we can see that the distributions are broader and different in shape than the structural ones, indicating that a direct relationship structure-function cannot be established. However, the same trend is maintained, with the tracks configuration showing higher CC values on average than the rest. Thus, we conclude that, first, effective connectivity cannot be freely taken as a proxy of the structural one, and second, that clustering coefficients (and by extension other topological properties) may by themselves not be sufficient to understand the observed rich dynamics in the topographical networks. Other traits may be necessary ingredients to render a full picture, such as strong anisotropies in the network's spatial connectivity (e.g., the distribution of connectivity angles in Figure 5), metric correlations, or connectivity motifs that amplify or restrain neuronal activity. Understanding the relative impact of all these ingredients is an endeavor that requires future substantial investigation.
